# Tregitopes and impaired antigen presentation: Drivers of the immunomodulatory effects of IVIg?

**DOI:** 10.1002/iid3.167

**Published:** 2017-05-31

**Authors:** Laetitia Sordé, Sebastian Spindeldreher, Ed Palmer, Anette Karle

**Affiliations:** ^1^ Novartis Pharma AG, Integrated Biologics Profiling Unit Immunogenicity Risk Assessment Basel Switzerland; ^2^ Novartis Institute for Biomedical Research, PK Sciences Biologics Basel Switzerland; ^3^ Department of Biomedicine, University Hospital Basel Transplantation Immunology and Nephrology Basel Switzerland

**Keywords:** Antigen presentation, IVIg, Tregitopes

## Abstract

**Introduction:**

Although intravenous immunoglobulin (IVIg) is commonly used in the clinic to treat various autoimmune and severe inflammatory diseases, the mode of action is not fully elucidated. This work investigates two proposed mechanisms: (1) the potential role of regulatory T‐cell epitopes (Tregitopes) from the constant domain of IgG in the immunosuppressive function of IVIg; and (2) a potential impact of IVIg on the ability of antigen presenting cells (APCs) to present peptides.

**Methods and Results:**

Investigation of the HLA class II peptide repertoire from IVIg‐loaded dendritic cells (DCs) via MHC‐associated peptide proteomics (MAPPs) revealed that numerous IgG‐derived peptides were strongly presented along the antibody sequence. Surprisingly, Tregitopes 167 and 289 did not show efficient natural presentation although they both bound to HLA class II when directly loaded as “naked” peptides on human DCs. In addition, both Tregitopes could not reproduce the inhibitory effect of IVIg in a human in vitro T‐cell proliferation assay as well as in vivo in mice. MAPPs data demonstrate that presentation of peptides from several antigens remained unchanged even when competed with high doses of IVIg, in both human and mouse.

**Conclusion:**

These data suggest that the effects mediated by IVIg are not caused by Tregitopes nor by impaired antigen presentation.

## Introduction

Intravenous immunoglobulin (IVIg) is a pool of plasma polyclonal IgG derived from thousands of healthy donors. Initially employed as a replacement therapy in patients suffering from immunodeficiency 1, it was discovered in 1980 that IVIg surprisingly ameliorated immune thrombocytopenia by immunosuppressive properties 2. Since then, the use of IVIg in the clinic to treat patients with a wide range of autoimmune and inflammatory disorders has considerably increased 3. IVIg is commonly administered at very high doses (1–4 g/kg) in order to achieve anti‐inflammatory effects. However, its mechanisms of action remain poorly understood and are still under debate.

Aberrant activation of the immune system is controlled by a subset of the T cell population, the regulatory T cells (Tregs) with suppressive activities ensuring peripheral self‐tolerance and immune homeostasis 4. Several studies highlight the possibility that IVIg might exert its immunosuppressive effects by inducing Tregs 5, 6. However, the events by which IVIg expands Tregs are not yet elucidated. De Groot et al. used in silico methods to identify conserved peptides in the constant regions of IgG that bind to multiple major histocompatibility complex (MHC) class II molecules 7. According to their studies, co‐administration of a specific antigen with these peptides suppressed immune response towards the antigen both in vitro in human peripheral blood mononuclear cells (PBMCs) and in vivo in mice. Intriguingly, addition of such peptides in vitro resulted in the expansion of Tregs accompanied by an increased secretion of IL‐10. Thus, they acknowledged these peptides as Treg epitopes, also named “Tregitopes.” According to this concept, MHC‐II: Tregitope complexes on antigen presenting cells (APCs) directly activate natural Tregs, which in turn convert the numerous neighboring effector T cells into adaptive Tregs, tipping the balance to immune tolerance. In this context, further re‐introduction of the immunogenic antigen in the absence of Tregitopes would still result in tolerization thanks to antigen‐specific adaptive Tregs. According to this model, the presence of Tregitopes in the constant regions of IgG would support the notion that IVIg might promote its anti‐inflammatory effects by activating Tregs.

While this concept implies that APCs efficiently co‐present both antigen‐derived peptides and IVIg‐derived Tregitopes on their surface, some other recent studies indicate immunosuppressive effects of IVIg being linked to an impaired antigen presentation capacity by APCs. Aubin et al. suggested that IVIg could directly compete with antigenic peptides for loading on MHC class II molecules 8, 9, 10, whereas others showed in contrast that IVIg decreased antigen uptake and processing 11, 12.

In light of such controversy in the field, we show to our knowledge for the first time the natural presentation of IVIg‐derived peptides via HLA/MHC class II on human and mouse APCs using MHC‐associated peptide proteomics (MAPPs). Despite Tregitopes bound to HLA‐DR when given as “naked” peptides, the results obtained indicate that Tregitopes 167 and 289 were not efficiently presented both in vitro and in vivo when full IgG processing was required. Moreover, we demonstrate that Tregitopes did not reduce an antigen‐specific immune response both in vitro in human PBMCs and in vivo in mice as compared to IVIg. Notably, MAPPs data indicate that antigen processing and presentation of peptides from several antigens is not impaired in the presence of high dose IVIg. Taken together, these results suggest that IVIg exerts its immunosuppressive effects via other mechanisms.

## Results

### Identification of HLA‐DR associated peptides naturally processed in vitro from IVIg

**Figure 1:**
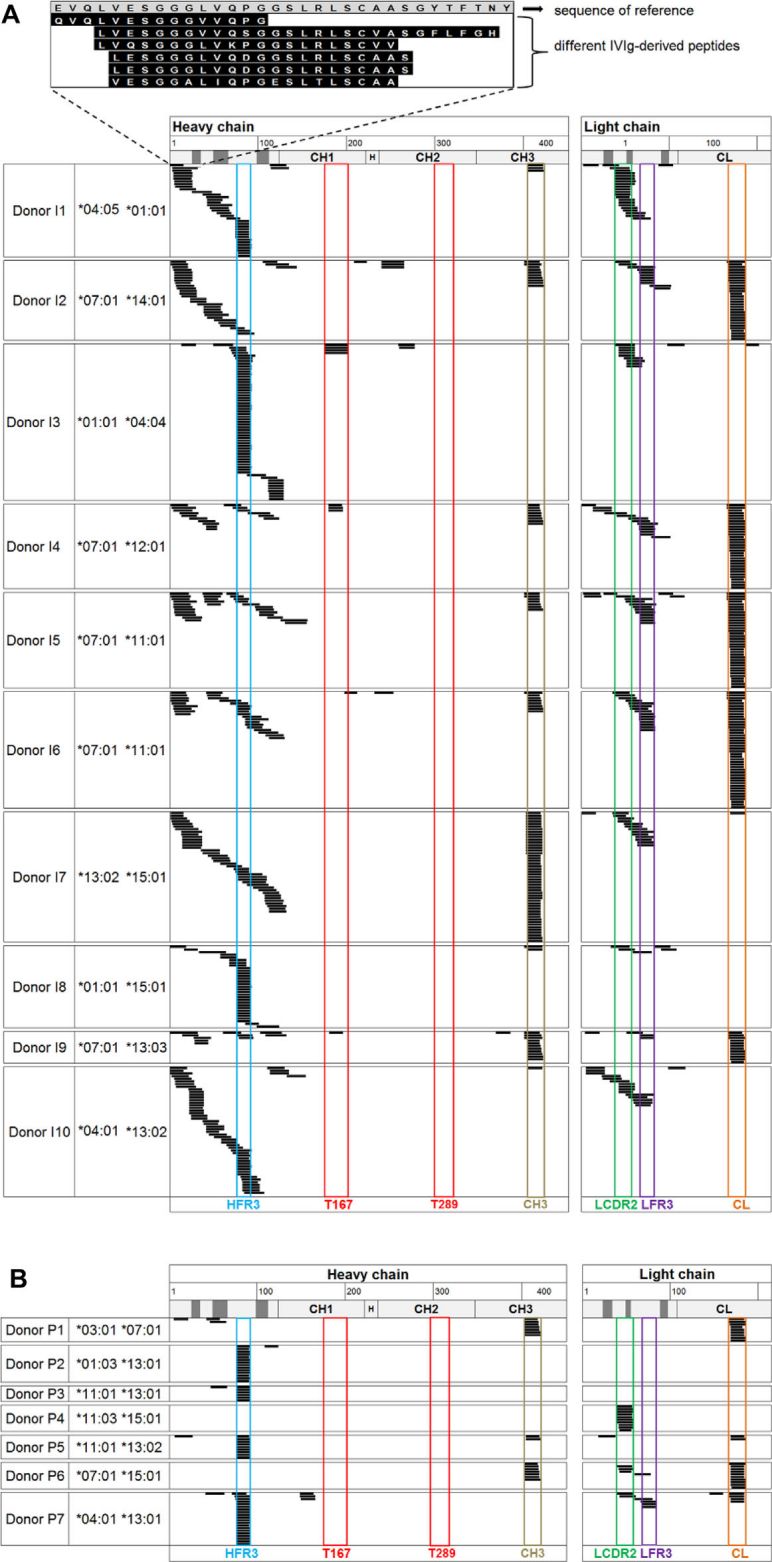
IVIg‐derived peptides naturally presented on HLA‐DR identified by MAPPs. (A) A total of 5.4 × 10^6^ DCs were loaded with IVIg at 2.5 pM. (B) HLA‐DR peptides derived from endogenous self‐IgG identified from 8 × 10^7^ fresh human PBMCs. Peptides were detected by LC‐MS multiple times and in several different length variants (black bars), forming clusters in several regions along the sequence of a human IgG_1_ antibody used for alignment. Only peptides sharing at least nine amino acids with the reference sequence were aligned. CDR regions are depicted in dark gray. Strongly presented sequence regions are highlighted and named according to their position in the molecule. Positions of Tregitopes (T) 167 and 289 are highlighted in red. H, heavy chain; L, light chain; FR, framework; C, constant region. HLA‐DRB1 alleles are indicated.

According to the in silico predictions performed by De Groot et al. 7, Tregitopes 167 and 289 are highly promiscuous binders of HLA class II molecules. We sought to evaluate the natural presentation of Tregitopes 167 and 289 in vitro via MAPPs, using liquid chromatography to separate the different HLA‐DR peptides in the sample and identify the eluted peptides by mass spectrometry and subsequent database search 13, 14, 15, 16, 17. MAPPs assay was applied on IVIg‐loaded human DCs. Monocyte‐derived DCs from 13 different donors were loaded with IVIg for 24 h (Fig. 1A). For the purpose of visualization, IVIg‐derived peptides identified via the MAPPs assay were aligned to the sequence of a human IgG_1_ antibody, which allowed for identification of sequence regions strongly presented by DCs. Exemplarily, data for IVIg‐loaded human DCs from 10 donors are shown. Only peptides sharing at least nine amino acids with the reference sequence are depicted. Therefore, it is possible that peptides either match entirely with the monoclonal antibody reference sequence or that they differ in several amino acids, although these differences did not prevent their alignment with the sequence of reference. Strikingly, numerous IVIg‐derived peptides were identified, clustering in several regions along the antibody sequence (complete list of identified peptides in Supporting Information Fig. S1). Identification of various length variants in each cluster is reflecting the ability of HLA class II molecules to bind peptides of variable lengths in the open‐ended peptide binding groove 18. Due to the polymorphic nature of the HLA‐DR locus in human and the resulting diversity in terms of binding properties of the different HLA‐DR molecules, the pattern of presented clusters differs considerably between the different donors. Several sequence regions showed promiscuous binding to different HLA‐DR alleles. The variable regions of both the heavy and light chains of IgGs contained in the IVIg preparation were strongly presented, even though peptides in the hypervariable regions could not always be mapped due to high sequence discrepancies relative to the reference antibody sequence. The peptides presented in the variable domains showed some sequence diversity, reflecting the sequence diversity in the IVIg preparation. This illustrates that several peptides differing by few amino acids are able to bind to the same HLA class II molecule owing to conserved binding residues. Among the presented regions along the IgG sequence, five strongly and commonly presented sequence regions were selected and named according to their position in the molecule: two in the variable region (LCDR2, LFR3) and one in the constant region of the light chain (CL) as well as one in the variable region (HFR3) and one in the constant region of the heavy chain (CH3), respectively, as highlighted in Figure 1A. The cluster presented in the light chain constant region (CL) occurred only in donors sharing the HLA‐DRB1 allele 07:01, indicating a highly restricted HLA‐DR allele binding specificity. Tregitope 167 only appeared in a small proportion of donors (3/13), whereas Tregitope 289 could not be detected in any of the tested donors.

To evaluate whether Tregitopes 167 and 289 may be presented more efficiently in vivo, fresh PBMCs isolated from buffy coats were analyzed by MAPPs. IgG‐derived peptides were detected from self‐antibodies naturally ingested by APCs present in the blood circulation (Fig. 1B). The peptide clusters identified were highly similar to those identified in DCs loaded in vitro with IVIg (complete list of identified peptides in Supporting Information Fig. S2). Neither Tregitope 167 nor Tregitope 289 was detected in peptide samples isolated from the fresh PBMCs.

Although many different IVIg‐derived peptides which were naturally presented on HLA‐DR were identified across multiple donors, little or no presentation of Tregitopes 167 and 289 was observed when processing was required.

### Tregitopes can be efficiently presented on HLA‐DR in human DCs when tested as “naked” peptides

**Figure 2:**
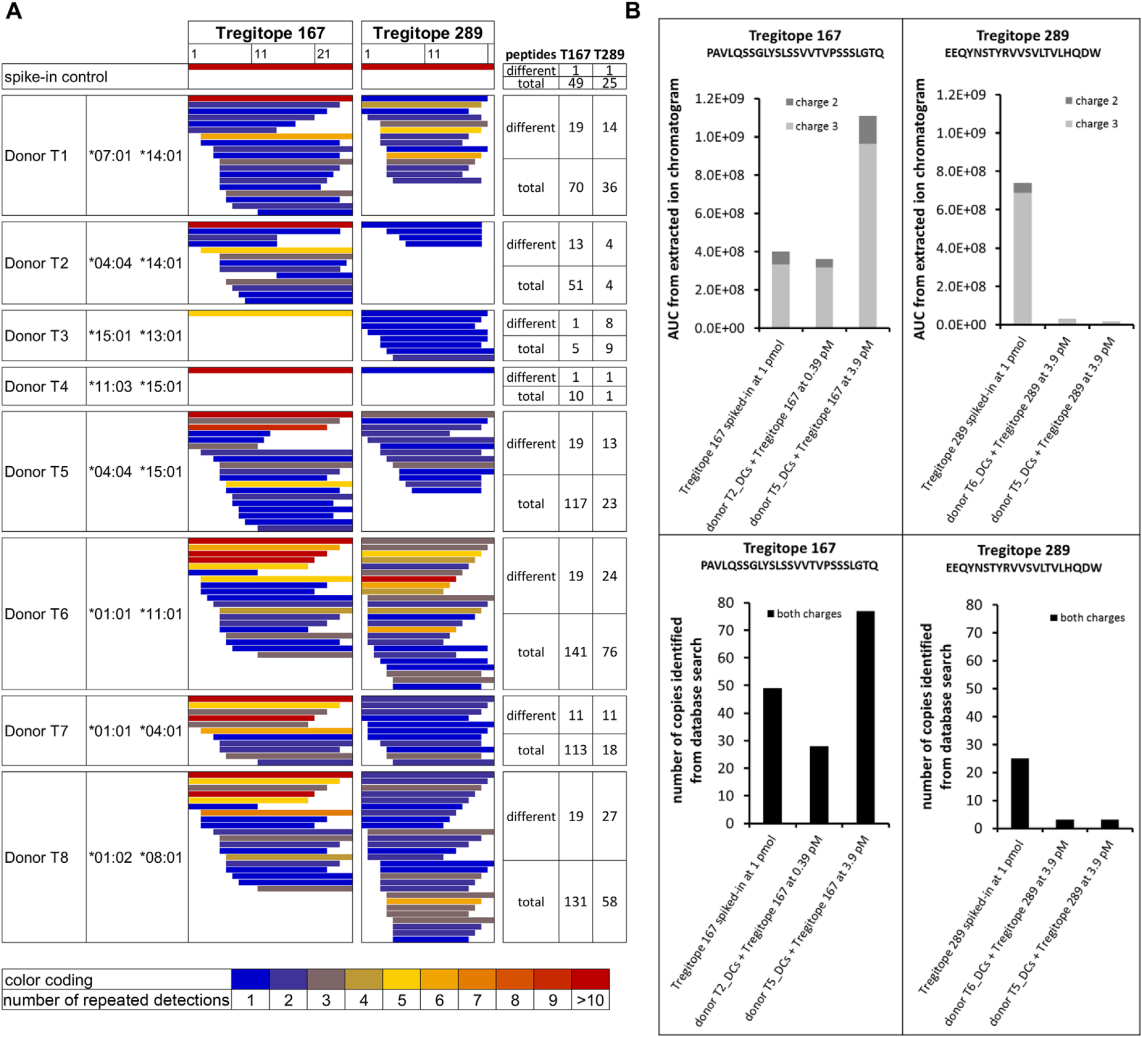
Tregitopes 167 and 289 HLA‐DR derived peptides identified via MAPPs from human DCs loaded with the “naked” Tregitope peptides. (A) For detection control, 1 pmol of each Tregitope was spiked into a HLA‐DR peptide sample isolated from mature DCs. A total of 5.4 × 10^6^ DCs from eight different donors were loaded with Tregitope at 0.39 pM (donors T1 to T3) or at 3.9 pM (donors T4 to T8). HLA‐DRB1 alleles are indicated for each donor. Tregitope‐derived peptides were quantified as “different” peptides including length variants with unique sequences and “total” peptides including repeated detections of the length variants. The peptide colors are reflecting the number of repeated detections. (B) Comparison of the quantification of full‐length Tregitopes 167 and 289 peptides via peak integration from the extracted ion chromatograms versus the number of peptides identified from the database search approach.

To rule out false negative results in the identification of Tregitopes from IVIg‐loaded DCs, efficient peptide detection via LC‐MS was confirmed and the presentation of Tregitopes loaded on DCs as “naked” peptides was evaluated. Each Tregitope peptide was individually spiked into a complex sample of HLA‐DR‐associated peptides which were isolated from mature DCs (Fig. 2A). Both Tregitopes could be identified as full‐length peptides in the spike‐in control, demonstrating efficient peptide reconstitution, and detection.

Next, monocyte‐derived DCs were loaded with the “naked” Tregitope peptides 167 or 289. Such peptides do not require further processing and therefore should result in surface loading of HLA‐DR molecules 19. In this setup, Tregitope peptides were efficiently presented by all of the eight donors tested, as indicated by repeated detections of mass spectra of both Tregitopes in several lengths variants (Fig. 2A). Peptide 167 was mostly identified in full length, while peptide 289 was often identified in shorter length variants, indicating significant proteolytic processing (Supporting Information Fig. S3). Although lysosomal degradation is assumed to be the usual pathway for antigen processing 20, there is also evidence that peptides from proteins degraded by extracellular proteases can directly bind to MHC class II on the surface of APCs 21. Therefore, the presentation of Tregitope length variants may either be due to intracellular processing after recycling of surface HLA‐DR molecules 22, 23 or due to trimming by extracellular proteases.

The full‐length Tregitope peptides 167 and 289 were quantified by two methods, based on the integration of the area under the peptide elution peak or based on the number of peptide identifications from the database search approach. For both peptides, the two quantitation approaches correlated well (Fig. 2B) and revealed markedly weaker presentation of full‐length Tregitope 289 as compared to Tregitope 167, which is consistent with the observed higher susceptibility of Tregitope 289 to degradation.

In line with the HLA‐DR binding results published by De Groot et al. 7, our data demonstrate that both Tregitopes 167 and 289 can efficiently bind to HLA class II, when tested as “naked” peptides.

### Tregitopes do not inhibit antigen‐specific human T‐cell proliferation in vitro

We evaluated the suppressive potency of published Tregitopes 167 and 289 on human T‐cell proliferation in vitro. Seven additional IgG sequence regions were tested, that showed strong presentation in the MAPPs assay across several human individuals (Table 1). Some of these peptides were derived from the constant region, similar to the published Tregitopes, while other peptides were selected from non‐conserved regions which should not act as Tregitopes based on De Groot et al.’ model. A peptide derived from the highly immunogenic major birch pollen allergen (Betv1a _108–126_) was synthesized as non‐IgG derived control peptide.

**Table 1:** List of peptides synthesized for testing in human T‐cell proliferation assay

	Name	Chain	Fragment	Peptide	Sequence of reference
Human peptides	*HFR3‐H*	Heavy chain	Fab	KNSLYLQMNSLRAEDTA	IgG*
	*HFR3‐R*	Heavy chain	Fab	KSAVYLQMTDLRTEDTG	IgG*
	T167	Heavy chain	Fc	PAVLQSSGLYSLSSVVTVPSSSLGTQ	De Groot et al.
	T289	Heavy chain	Fc	EEQYNSTYRVVSVLTVLHQDW	De Groot et al.
	LCDR2	Light chain	Fab	LPGTAPKLLIYSNNQRPSG	IgG*
	*LCDR2‐H*	Light chain	Fab	KPGKAPKLLIYAASTLQSG	IgG*
	LFR3	Light chain	Fab	GSGTDFTLTLSRLEPED	IgG*
	*LFR3‐H*	Light chain	Fab	GSGTDFTLTISSLQPED	IgG*
	CL	Light chain	FC	DSKDSTYSLSSTLTLSKA	IgG
Irrelevant peptide	Be tv 1a	Position 108‐126		TPDGGSILKISNKYHTKGDH	Major birch pollen allergen

T, Tregitope; C, constant region; FR, framework region; H, heavy chain; L, light chain; CDR, complementarity‐determining region. Sequences of Tregitopes and well‐presented IgG‐derived peptides identified by MAPPs are listed. For peptides in the variable regions showing high sequence diversity, one sequence was selected for testing in human in vitro T‐cell assay. Peptides are named according to their position in the antibody molecule. *Indicates that the peptide listed was identified in several variations differing by a few amino acids.

**Figure 3:**
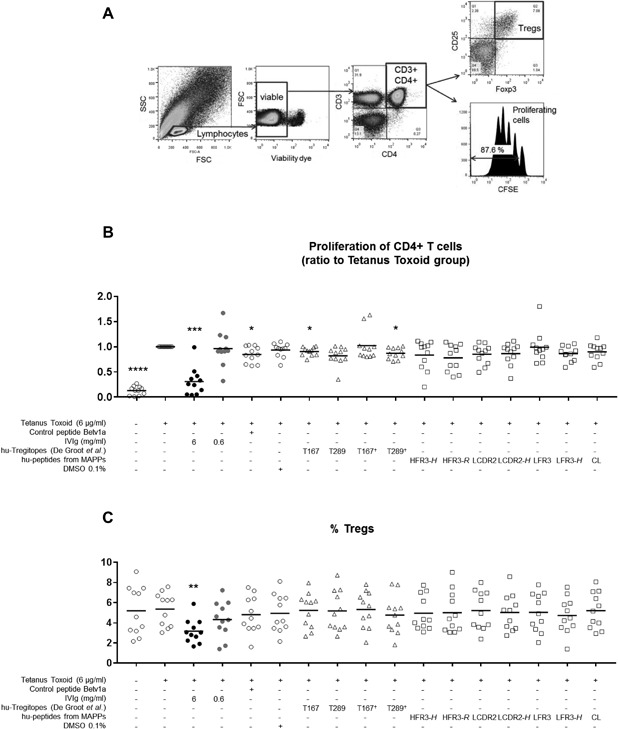
Tregitopes and well presented peptides identified by MAPPs have no inhibitory effect on proliferation of human CD4+ T cells stimulated with tetanus toxoid. Human PBMCs were stimulated with 6 µg/mL of tetanus toxoid (TT) and peptides were added simultaneously at 10 µg/mL (3.9 pM). (A) Gating strategy for flow cytometry. Tregs were defined as CD3 + CD4 + CD25 + Foxp3+. (B) Proliferation of CD4+ T cells after 7 days as measured by flow cytometry via CFSE incorporation. Values are expressed as ratio to the TT group. (C) % Tregs after 7 days. For both graphs black lines represent mean. *P < 0.05; **P < 0.01; ***P < 0.001; ^****^ *P* < 0.0001 (paired one‐way ANOVA, Dunnett's test, comparison to tetanus toxoid group). *n* = 11. Data are from five independent experiments. hu, human; †, acetylated and amidated peptide.

In the human T‐cell proliferation assay, each tested peptide was added simultaneously with tetanus toxoid (TT) to human PBMCs isolated from 11 donors, and CD4+ T‐cell proliferation was assessed after 7 days by flow cytometry via CFSE incorporation (Fig. 3A). T‐cell functionality was tested with PHA and strong responses were observed in all donors (data not shown). Proliferation results were normalized against the group stimulated with tetanus toxoid alone (Fig. 3B). Proliferation after stimulation with TT was on average 7.80‐fold higher as compared to unstimulated cells. Addition of human Tregitopes 167 or human Tregitope 289 chemically modified (N‐acetylated and C‐amidated) slightly but significantly reduced T‐cell proliferation by 1.10‐ and 1.15‐fold, respectively. However, addition of the control peptide Betv1a together with TT also significantly reduced T‐cell proliferation by 1.18‐fold. Therefore, the decrease in T‐cell proliferation observed for these two Tregitopes was not peptide‐specific and considered as null. Neither of the other tested peptides from both IgG constant and variable regions was able to inhibit T‐cell proliferation. Interestingly, IVIg used at 6 mg/mL led to a significant reduction of T‐cell proliferation of 3.24‐fold, while this inhibitory effect was not observed at a dose 10 times lower.

In the same set of experiments, the population of Tregs was investigated (Fig. 3C). In contrast to the results published by De Groot et al., co‐incubation with Tregitopes 167 and 289 (both chemically modified and non‐modified) did not lead to expansion of Tregs after 7 days of stimulation. The other test peptides also showed no effect on the activation of Tregs. However, high doses of IVIg significantly decreased the proportion of Tregs (2.01%) in comparison to the unstimulated control.

These data demonstrate that in our hands, Tregitopes did neither inhibit antigen‐specific human T‐cell proliferation nor expand the population of Tregs in vitro.

### Tregitopes are not efficiently presented on MHC class II in mice and do not induce tolerance in vivo

**Figure 4:**
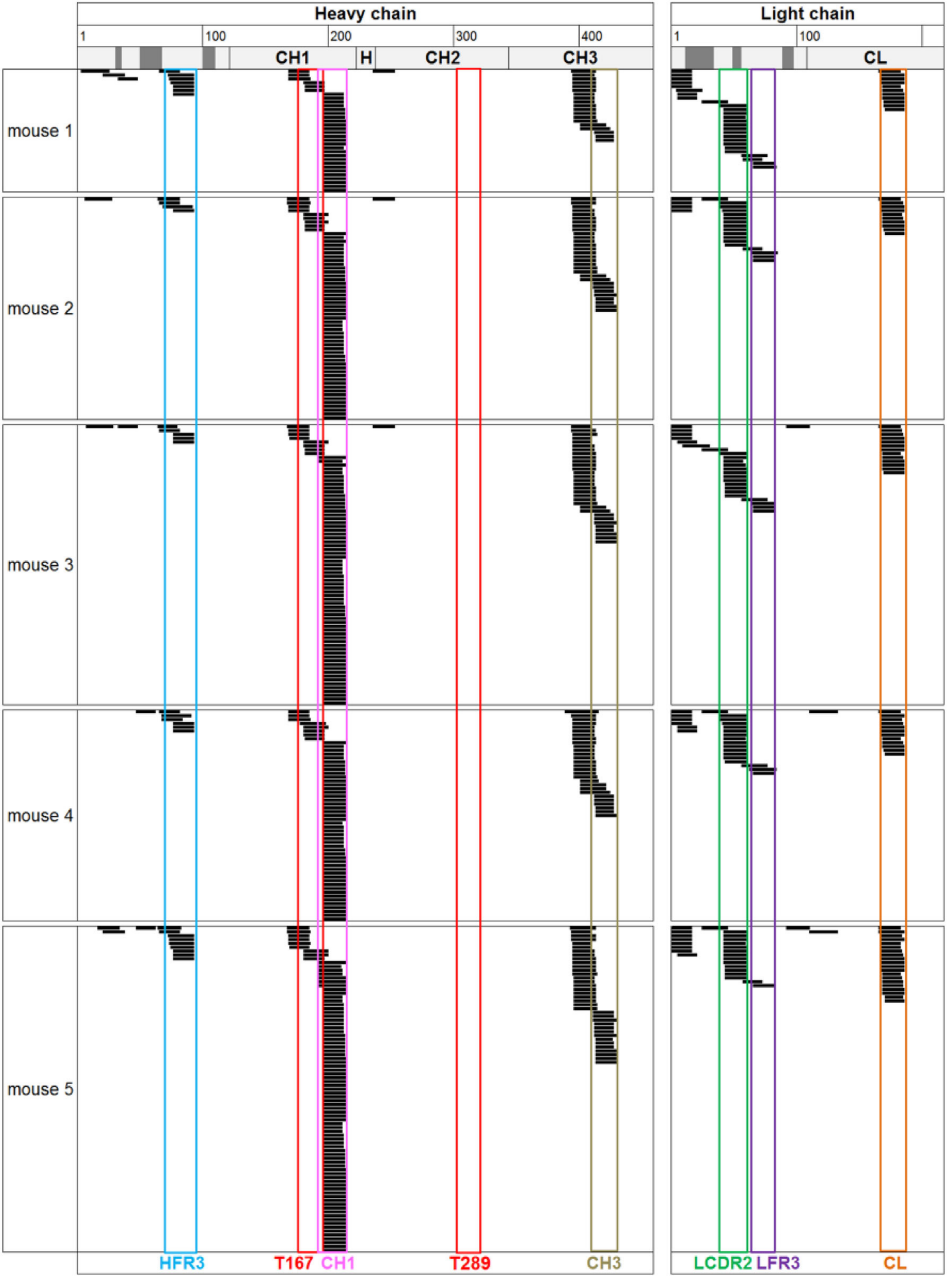
Naturally presented MHC class II peptides derived from endogenous IgG identified from C57Bl/6 mouse splenocytes. Peptides isolated from half spleens were detected by LC‐MS multiple times and in several different length variants (black bars), forming clusters in several regions along the sequence of a mouse IgG_2a_ antibody used for alignement. Only peptides sharing at least nine amino acids with the reference sequence were aligned. CDR regions are depicted in dark gray. Strongly presented sequence regions are highlighted and named according to their position in the molecule. T, Tregitopes; H, heavy chain; L, light chain, FR, framework; C, constant region.

To explore whether Tregitopes show a more efficient presentation in vivo, naturally presented peptides were isolated from the APCs from spleens of C57Bl/6 mice followed by MAPPs (Fig. 4). The cluster pattern of mouse IgG‐derived peptides presented on MHC class II corresponded remarkably well to the pattern established from human DCs loaded with IVIg in vitro and from fresh human PBMCs that had engulfed IgG from the blood stream in vivo (complete list of identified peptides in Supporting Information Fig. S4). Compared to human, mice presented two more dominant clusters located in the CH1 and CH3 domains. The cluster pattern was highly similar between the five different animals tested as all mice were genetically identical (inbred strain) and consequently homozygous at all loci including their MHC class II receptor (I‐A^b^). These results also underline the reproducibility of the MAPPs assay. The mouse equivalent to Tregitope 167 could not be identified in full length. Instead two overlapping clusters were identified, covering the Tregitope sequence only partially, either C‐ or N‐terminally. In concordance to our findings from human IVIg‐loaded DCs, mouse Tregitope 289 could not be identified in any mouse.

**Figure 5:**
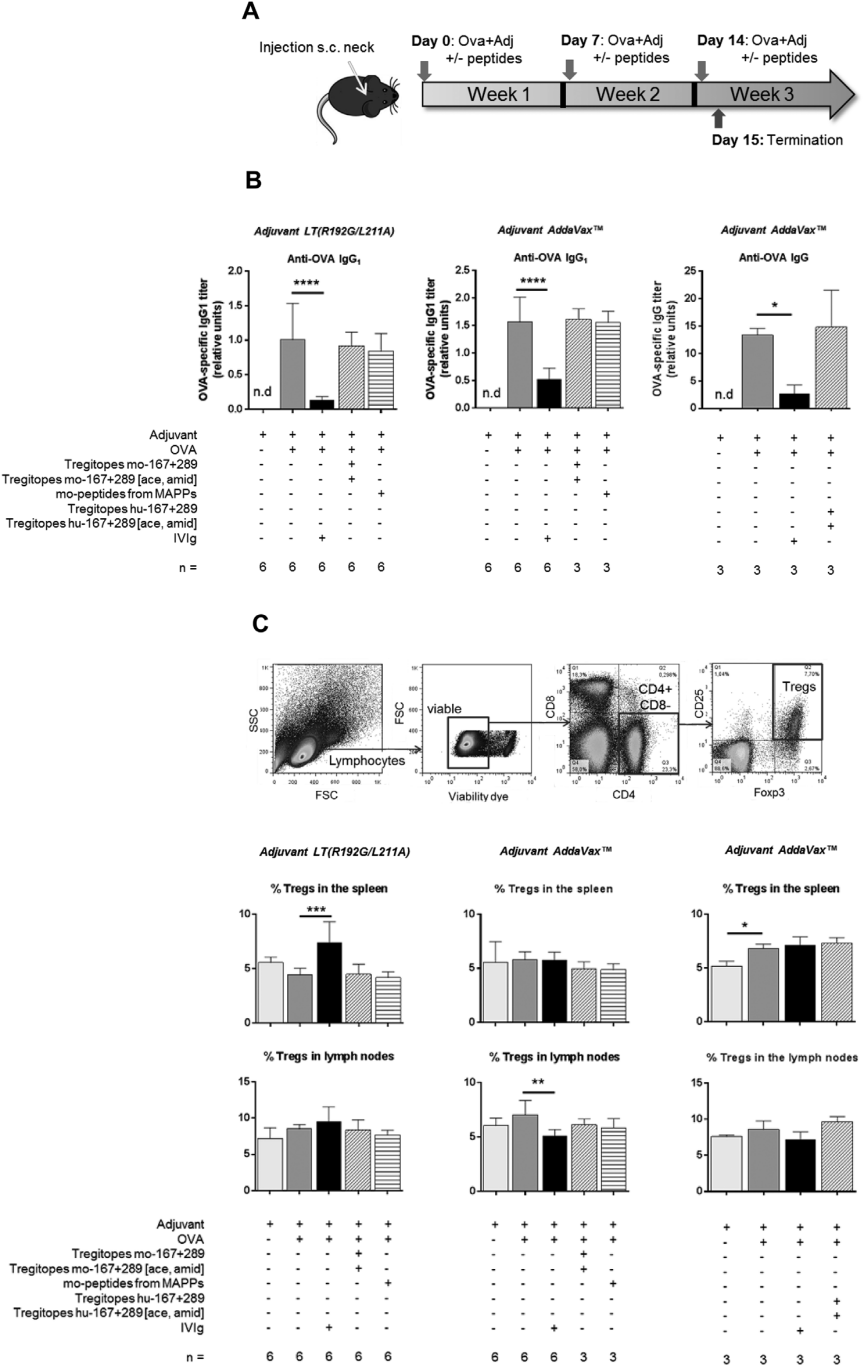
Tregitopes and well presented peptides identified by MAPPs did not reduce OVA‐specific antibody response in mice. (A) C57Bl/6 mice were injected three times weekly s.c in the neck with 50 µg OVA and adjuvant, and co‐injected with or without peptides (50 µg each) or IVIg (50 mg). Mice were terminated 24 h after last injection. (B) Anti‐OVA IgG were measured by ELISA. (C) % Tregs in spleen or in draining lymph nodes was determined by flow cytometry. Tregs were defined as CD4 + CD25 + Foxp3+. n indicates the number of different mice tested. Mean values ± SD are reported from a single experiment. *P < 0.05; **P < 0.01; ***P < 0.001; ^****^ *P* < 0.0001 (one‐way ANOVA, Dunnett's test, comparison to’ Adjuvant + OVA’ group). mo, mouse; hu, human.

To evaluate the inhibitory properties of Tregitopes in vivo, C57Bl/6 mice were immunized with Ovalbumin (OVA) (Fig. 5A), and the test peptides (Table 2) were co‐injected according to the protocol published by De Groot et al. 7. Consistent with our human in vitro data, neither the mouse Tregitopes 167 and 289 nor any other tested mouse‐derived peptides from well‐presented IgG sequence regions identified by MAPPs were able to downregulate the production of OVA‐specific antibodies in mouse serum (Fig. 5B). Importantly, co‐injection with adjuvant LT_(R192G/L211A)_ or adjuvant MF59 (AddaVax^®^) led to similar results. Co‐administration of OVA together with human sequences of Tregitopes 289 and 167 was tested, and in line with their mouse counterparts they did not suppress the antibody response against OVA (Fig. 5B). In contrast, injections of high doses of IVIg (50 mg/animal, which is similar to the clinical dose) led to a strong and significant reduction of OVA‐specific antibodies in all experiments. No significant difference in the proportion of Tregs was observed upon administration of Tregitopes or other test peptides (Fig. 5C).

**Table 2:** List of peptides synthesized for testing in mouse in vivo assay

	Name	Chain	Fragment	Peptide	Sequence of reference
Mouse peptides	T167	Heavy chain	Fc	PAVLQSDLYTLSSSVTVPSS	De Groot et al.
	CHI	Heavy chain	Fc	VPSSTWPSQTVTCNVAHPASSTK	IgG
	T289	Heavy chain	Fc	EEQFNSTFRSVSELPIMHQ	De Groot et al.
	CH3	Heavy chain	Fc	DTDGSYFVYSKLNVQKSNWEA	IgG
	LCDR2	Light chain	Fab	KPDGTVKLLIYYTSRIHSGVPS	IgG*
	LFR3	Light chain	Fab	GSGSGRDYSFSLSNLEPED	IgG*
	CL	Light chain	Fc	DQDSKDSTYSMSSTLTLTKD	IgG

T, Tregitope; C, constant region; FR, framework region; H, heavy chain; L, light chain; CDR, complementarity‐determining region. Sequences of Tregitopes and well‐presented IgG‐derived peptides identified by MAPPs are listed. Peptides are named according to their position in the antibody molecule. *Indicates that the peptide listed was identified in several variations differing by a few amino acids.

We conclude that Tregitopes 167 and 289 are naturally not well presented on MHC class II in vivo in mice and that neither Tregitopes nor IgG‐derived well‐presented peptides possess an immunosuppressive activity.

### IVIg does not impair antigen presentation on MHC class II molecules both in vitro and in vivo

**Figure 6:**
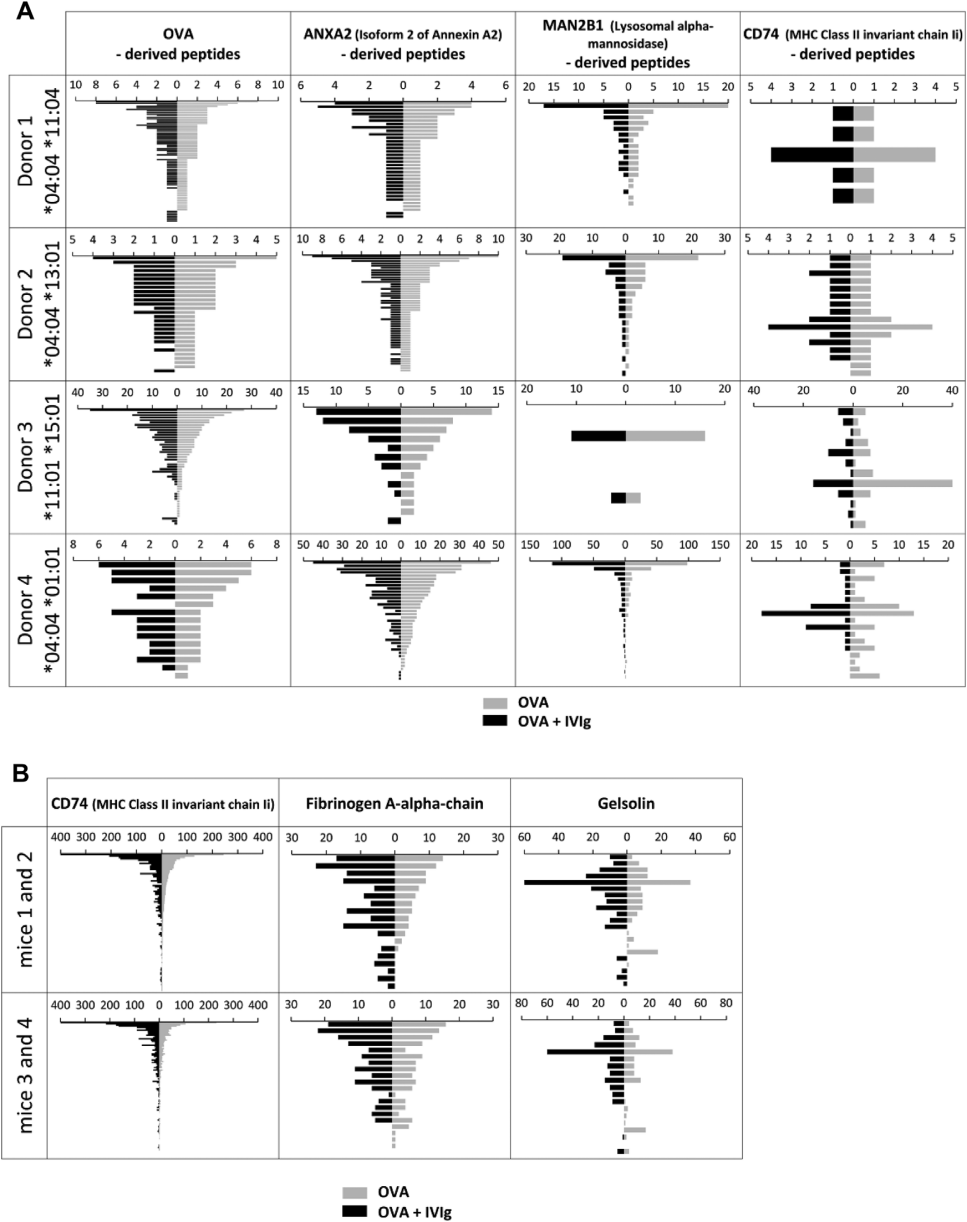
Co‐administration of high dose IVIg does not impair HLA‐DR and MHC class II antigen presentation. Each unique peptide sequence identified via the database search approach is depicted for both conditions OVA + IVIg (dark gray) and OVA alone (light gray) ranked from the most abundant peptides to the least abundant peptides in the OVA group. The size of each bar indicates the number of detection repetitions for each sequence. (A) MAPPs data from human DCs loaded with OVA with or without IVIg.OVA was added at 14 nmol/mL. IVIg was added at 14 nmol/mL (2 mg/mL) for donor 1–3 and at 117 nmol/mL (17 mg/mL) for donor 4. OVA‐derived peptides are shown in the left column, and peptides from three other well‐presented proteins, ANXA2, MAN2B1, and MHC class II invariant chain Ii (CD74) are depicted in the midle and right column, respectively. (B) MAPPs data from half spleen isolated from C57Bl/6 mice immunized with OVA and adjuvant with or without IVIg. Mice were injected three times weekly s.c in the neck with 50 µg OVA and adjuvant, with or without IVIg (50 mg), and terminated 24 h after last injection. Peptides from abundantly presented endogenous proteins, MHC class II invariant chain Ii (CD74), Fibrinogen A‐alpha chain and Gelsolin, are shown in the left, middle, and right column, respectively.

Having demonstrated that IVIg is processed to a multitude of peptides presented on HLA‐DR, we sought to investigate whether IVIg could impair antigen presentation. For this purpose, human DCs were co‐loaded with OVA and IVIg in vitro, and HLA‐DR‐associated peptides were identified via MAPPs. Peptide presentation was semi‐quantitated via the number of repeated identifications of each peptide via the database search approach (Fig. 6A) and confirmed via the peak integration method (Supporting information Fig. S5A and B). In four different donors tested, the number of OVA‐derived peptides presented on HLA‐DR was similar in DCs samples loaded with or without IVIg. Similarly, IVIg did not impair the presentation of peptides derived from two endogenous self‐proteins, isoform of annexin A2 (ANXA2), and lysosomal alpha‐mannosidase (MAN2B1) which were abundantly presented by all donors.

To examine the influence of IVIg on antigen presentation in vivo (Fig. 6B), mice were administered with OVA and adjuvant with or without IVIg, and MHC class II peptides were isolated from the spleen and analyzed via MAPPs. Due to the low dose of OVA administered, OVA‐derived peptides could not be detected in spleen samples. Peptides derived from three abundantly presented endogenous proteins, namely the MHC class II invariant chain Ii (also known as CD74), the Fibrinogen A‐alpha chain (FGA), and the Gelsolin protein (GSN) however, could be used to evaluate the impact of IVIg co‐administration on their peptide presentation levels. Data were consistent among the two pairs of mice tested, and revealed that co‐administration of doses of IVIg in the same range as clinically administered doses of IVIg had no effect on the peptide‐presentation of these proteins. The number of peptide copies identified for CD74 was very high in all mice (up to 400) in comparison with the two other proteins (maximum 20 and 60 copies for Fibrinogen A‐alpha chain and Gelsolin, respectively). This is consistent with the presence of abundant MHC class II‐Ii complexes in the endosomes of APCs, and the fact that the class II‐associated invariant chain peptide (CLIP) remains associated within the binding groove if no binding of antigenic peptides occurs 21. As the number of CD74‐derived peptides was similarly high with and without the co‐administration of IVIg, this reveals that there were still a lot of free MHC class II molecules available for antigen binding, and that the maximum capacity of antigen presenting cells was not reached even with high dose of IVIg.

Overall these data indicate that HLA class II and MHC class II antigen presentation is not affected by co‐administration of high doses of IVIg, both in vitro and in vivo.

## Discussion

Over the past years, researches have been investigating the therapeutic mode of action of IVIg 24, 25. Several mechanisms have been proposed, among which De Groot et al. 7 have described that the presence of Tregitopes 167 and 289 in the constant region of IVIg would account for its immunomodulatory properties.

Here, we have investigated to our knowledge for the first time the natural presentation of IVIg‐derived sequences by isolating HLA‐DR‐associated peptides from human DCs loaded with IVIg in vitro. Interestingly, several IgG‐derived sequence regions were strongly presented, particularly in the variable domains. This is line with a recent study showing that sequences in immunoglobulin heavy chain variable region are enriched for binding to MHC molecules 26. Surprisingly, Tregitopes were able to be efficiently presented by antigen presenting cells only when tested as “naked” peptides, but not when processing of whole IgG molecules was required. Consistently, the repertoire of naturally presented self‐IgG derived‐peptides from fresh PBMCs derived from healthy donors was comprising several IgG‐derived‐peptides, but not the Tregitope peptides 167 and 289. Similarly, Tregitopes 167 and 289 could not be identified among the naturally presented self‐IgG derived‐peptides isolated from mouse spleens, although numerous peptides clustering in the constant regions of the heavy chain were identified. This finding is in agreement with previous experiments confirming the presentation of peptides derived from the constant heavy chain domains of IgG2a and IgG2ab molecules on the MHC class II in mice 27, 28, 29.

The finding that the Tregitopes can be efficiently presented when tested as “naked” peptides is consistent with the in silico predictions and HLA‐binding assay performed in the study of De Groot et al. In that case, no processing is required since the peptides can directly bind to HLA class II molecules. However, there is a clear discrepancy between the efficient presentation of the “naked” Tregitope peptides as opposed to the inefficient presentation within a full antibody molecule both in humans and mice. This strongly suggests that during the processing of IgGs in the endolysosome, Tregitope peptides 167 and 289 are either not efficiently generated or too readily degraded. On one side, relative resistance of Ig to processing has been demonstrated in a mouse tumor model showing that free light chains were much more efficient than complete Ig molecules in stimulating idiotype‐specific T cells 30, which is in line with similar findings for “preprocessed” Ig fragments 31. On the other side, the concept of rapid degradation is supported by the finding that after loading of DCs with the “naked” peptides 167 and 289, both peptides were degraded as indicated by the detection of several shorter length variants, while peptide 289 was more heavily degraded than peptide 167. Independent of the mechanism leading to reduced presentation, these findings question the presumed role of Tregitopes in tolerance induction by IVIg, since induction of natural Tregs would require efficient presentation of Tregitope peptides in the context of HLA/MHC class II. This is consistent with data which we recently published 32: in that study, MAPPs was applied to identify HLA‐DR derived peptides from five marketed monoclonal antibodies. Although identification of Tregitopes was not the focus of this study, the peptides map reveals that Tregitope 167 was identified in 2 out of 10 donors only, whereas Tregitope 289 was not presented in any of the 10 donors for all five antibodies which were tested.

Interestingly, the MAPPs data presented here reveal strong similarities in the presented peptide pattern between human individuals and C57Bl/6 mice, underlining marked structural conservation of the MHC class II peptide‐binding pockets as well as IgG sequences during evolution.

To investigate the capacity of Tregitope peptides to down‐regulate the T‐cell response against a co‐injected antigen, T‐cell proliferation was measured in a human in vitro T‐cell assay. Tregitope peptides, IVIg, as well as several peptides identified from IVIg or mouse IgG were tested in combination with the antigen TT in a human PBMC assay. While IVIg showed a strong suppression on T‐cell proliferation against the antigen TT, loading of human PBMCs with Tregitopes 167 and 289 did not induce inhibitory effects on T‐cell proliferation. In the OVA immunization mouse model, Tregitopes 167 and 289 and strongly presented IVIg‐derived peptides were co‐injected with OVA in mice, and in concordance with human in vitro experiments, the Tregitopes did not down‐regulate the anti‐OVA IgG response in vivo. These results are in line with a recent study showing no significant remissions in non‐obese diabetic (NOD) mice treated with Tregitopes 33. As expected, peptides derived from non‐conserved IgG variable regions showed neither a suppressive effect on T‐cell proliferation in vitro nor on the anti‐OVA‐IgG response in vivo. Surprisingly, strongly presented MHC class II peptides derived from IgG constant regions equally failed to suppress effector immune responses against a specific antigen in both human and mouse experiments, putting the Tregitopes concept to question. Moreover, the Tregitopes model, teaching the immune system to tolerate a given antigen by co‐delivering it with IVIg or Tregitope peptides, implies that further re‐administration of this antigen alone should not induce an immune response. In contrast, it is well established that IVIg does not cure but rather ameliorate most autoimmune and inflammatory diseases, since monthly injections are required over years. The fact that 87.5% of chronic inflammatory demyelinating polyneuropathy (CIPD) patients improved upon IVIg therapy, whereas 85.7% of the responsive patients worsened only a few months after IVIg discontinuation 34 indicates that IVIg does not establish tolerance in the classical sense.

Among the numerous proposed modes of action of IVIg, Aubin et al. proposed that the suppressive effect of IVIg on T cells might be an indirect consequence of a competition on the peptide presentation level and reduction in the antigen presentation ability of APCs 10. Here, we examined potential competition for antigen presentation by directly interrogating the natural presentation of HLA/MHC class II associated peptides using the MAPPs assay. Human DCs loaded in vitro with OVA with and without IVIg showed that presentation of OVA‐derived peptides remained unchanged even when competed with high doses of IVIg. Also the presentation of peptide derived from endogenous proteins was not affected. Similarly, in vivo OVA/IVIg co‐treatment of mice did not impact the peptide presentation of endogenous proteins. This demonstrates that IVIg did not compete with other proteins for processing by proteases and binding to HLA class II molecules in the endolysosomal compartment. Considering that antigen uptake in DCs occurs in a limited time‐window after maturation induction 22, 35, our results indicate that a sufficient number of HLA class II molecules are available to accommodate the peptides generated from all the proteins taken up during this time window. In line with our findings, several studies demonstrated that inhibition of T‐cell responses by IVIg does not result from diminished presentation of peptides on MHC class II 36, 37.

In summary, this work addressed the role of Tregitopes in the immunosuppressive properties of IVIg, and evaluated the ability of IVIg to influence peptide presentation on HLA/MHC class II molecules. Here, we have demonstrated that Tregitopes were not well presented when processing of full IgG antibody molecule was required and in our hands, Tregitopes were not able to dampen the immune response against a specific antigen neither in vitro nor in vivo. Moreover, we have shown that presentation of IVIg‐derived peptides did not alter the peptide presentation of other proteins in vitro and in vivo. Our data therefore suggest that immunomodulatory effects of IVIg are not mediated by Tregitopes or impaired antigen presentation. Intriguingly, MAPPs data revealed strong presentation of many different IVIg‐derived peptides, especially from the hypervariable regions. Although these peptides are most likely recognized as foreign and highly immunogenic 38, 39, 40, surprisingly IVIg efficiently inhibited antigen‐specific immune response across all experiments. Whether the presentation of these foreign peptides plays a role in IVIg immunosuppressive properties will require further investigations.

## Materials and Methods

### MHC associated peptide proteomics assay (MAPPs assay)

PBMCs were isolated from human buffy coats within 24 h after blood was sampled from consent healthy donors (Red Cross, Bern) according to local ethical practice. Isolation of HLA‐DR associated peptides from human monocytes‐derived DCs was performed as previously described 13, 32. For mouse samples, monoclonal antibodies generated using the mouse hybridoma cell line Y‐3P were used for the immunoprecipitation of MHC class II molecules. For isolation of MHC class II associated peptides, spleens collected from C57Bl/6NCrl mice (Charles River) were individually disrupted with a polytron in the same lysis buffer used for human samples. Peptide identification was performed by liquid chromatography‐electrospray ionization‐mass spectrometry as previously described 13. A database was created by combining either the human IPI (International Protein Index) v3.70 or the mouse IPI v3.87 with the specie‐related NCBI (National Center for Biotechnology Information) Ig V gene sequence database. Peptides were identified using a database search approach via the SEQUEST algorithm. The applied LC‐MS method is semi‐quantitative. Elution of a given peptide peak from the chromatography column will trigger multiple mass spectra. The chromatographic co‐elution of several peptide species at the same time, however, will compete with the detection in the mass spectrometer. Only high quality mass spectra can be adequately identified by database search. The NCBI database is only covering a limited portion of the possible IgG human repertoire and therefore, some mass spectra may not have been identified as IgG derived peptide, especially in the hypervariable regions. Using a sequence alignment tool, human‐derived peptides were aligned to an antibody sequence which is very close to germline, the antibody sequence of adalimumab (AbbVie). Mouse‐derived peptides were aligned to the antibody sequence of Muromonab‐CD3 (Janssen‐Cilag). Only peptides sequences with at least nine amino acids identical to the reference sequence are shown in the alignment. In addition, proper sequence alignment was manually confirmed and improperly aligned sequences were removed.

### Peptides synthesis

MAPPs selected peptides as well as Tregitopes 289 and 167 were synthesized by ProImmune (Oxford, UK) with a purity >90%. Lyophilized peptides were all dissolved in DMSO at 10 mg/mL followed by sonication for 20 min, and stored at −80°C.

### Human T‐cell proliferation assay

Human PBMCs were isolated from buffy coats as described in the previous section and labelled with 1 µM CFSE (Invitrogen, Carlsbad, CA, USA) at a concentration of 1 E^6^ cells/mL in pre‐warmed RPMI (Gibco Invitrogen) for 10 min at 37°C. Cells were washed, resuspended in X‐Vivo 15 medium (Lonza, Basel, Switzerland) supplemented with 10% FCS (Invitrogen) and 1% Glutamax (Invitrogen), and distributed in a 12‐well plate (Corning, New York City, NY, USA) at 5 E^6^ cells/mL per well. Tetanus Toxoid (TT) (Novartis, Marburg, Germany) was added to a final concentration of 6 µg/mL per well. IVIg (Octagam 10%; Octapharma, Lachen, Switzerland) was tested at 6 mg/mL (equivalent to 40 nmol/mL) or 0.6 mg/mL (equivalent to 4 nmol/mL). Human Tregitopes 289 or 167, peptides identified by MAPPs or the allergen birch pollen Betv1a peptide (TPDGGSILKISNKYHTKGDH) were added to the wells at 10 µg/mL (equivalent to 4 nmol/mL) and DMSO final concentration was 0.1% v/v. PHA (Sigma–Aldrich, Saint‐Louis, MO, USA) was used as a positive control for T‐cell proliferation and was added to a final concentration of 1 µg/mL per well. Unstimulated cells served as negative control. Cells were incubated at 37°C and 24 h later 1 mL of fresh complete X‐Vivo 15 medium was added on top of each well. Cells were harvested on day 7.

### Mouse experiments

#### Mice

All animal experiments were performed in accordance with the Federal and Cantonal laws of Switzerland. Animal protocols were approved by the Cantonal Veterinary Office of Basel‐Stadt, Switzerland. Female mice C57Bl/6NCrl were obtained from Charles River, France. All mice were used at an age of 7–11 weeks.

#### Mice immunization

Mice were immunized three times once a week sub‐cutaneously in the neck with 50 µg OVA/mouse (Invivogen, San Diego, CA, USA) together with adjuvant AddaVax™ (MF59, Invivogen) at 1:5 v/v in 600 µL DPBS or adjuvant LT (R192G/L211A) (kindly provided by Prof. J. D. Clements, Tulane University School of Medicine, USA) at 5 µg/mouse in 500 µL DPBS. When indicated, IVIg (Octagam 10%; Octapharma) was co‐injected in the same solution at 50 mg/mouse, or Tregitope peptides 7 or peptides identified via MAPPs assay at 50 µg/mouse each. Mice were terminated 24 h after the last injection.

#### Necropsy and organs processing

Blood was collected via the retro‐orbital path and serum was conserved at −20°C. Spleen and lymph nodes were dissected out and cell suspension was obtained by disrupting organs through a 40 µm nylon cell strainer (BD, Franklin Lakes, NJ, USA). In spleen samples, red blood cells were lysed for 5 min at room temperature (lysis buffer, Sigma). Cells were resuspended in complete Iscove's media (IMDM, 1% Glutamax, 10% FCS, Invitrogen; 1% penicillin/streptomycin, 50 μM Beta‐mercaptoethanol, 1% MEM non‐essential amino acids, 1 mM sodium‐pyruvate, Invitrogen).

### Antibody staining and flow cytometry

Fc‐gamma receptors were blocked by incubating human cells with 200 µg/mL of IVIg (Octagam; Octapharma), and mouse samples were incubated with a rat anti‐mouse CD16/CD32 antibody (BD clone 2.4G2) diluted 1:100. Human samples were stained with the following antibodies (final concentration in the well is indicated for each antibody): CD3‐Brilliant Violet 510 1:100 (BioLegend, San Diego, CA, USA, clone OKT3), CD4‐APC‐CY7 1:100 (BD Pharmingen clone RPA‐T4), CD25‐APC 1:100 (BD Pharmingen clone 2A3) Foxp3‐PE 1:50 (eBioscience, San Diego, CA, USA clone 236A/E7). Mouse‐specific antibodies were the following: CD3‐Brilliant violet 510 1:50 (BioLegend clone 17A2), CD4‐APC‐Cy7 1:100 (BD Pharmingen clone GK1.5), CD8α‐PE‐Cy7 1:100 (eBioscience clone 53‐6.7), CD25‐PercP Cy5.5 1:100 (BioLegend clone 3C7), FoxP3‐FITC 1:50 (eBioscience clone FJK‐16s). Dead cells were excluded using the fixable viability dye‐eFluor 450 1:1000 (eBioscience), and intracellular staining was performed using the fixation/permeabilization buffer set from eBioscience). Flow cytometry measurements were performed on a FACS Canto II instrument (BD) and the data were analyzed with FlowJo software (Tree Star).

### ELISA anti‐OVA IgG

Mouse OVA‐specific IgG1 (Cayman, Ann Arbor, MI, USA) and total IgG (Chondrex, Redmond, WA, USA) antibodies were measured following the instructions provided by the manufacturer's kits. OD was acquired at a wavelength of 450 nm on a Versa max reader (Molecular Devices, Sunnyvale, CA, USA). Results were analyzed on the Softmax^®^ Pro software (Molecular Devices) and data expressed as relative units compared to standard.

### Statistics

Graphs and statistical analyses were generated with GraphPad Prism. Paired *t*‐tests and one‐way ANOVA were performed where indicated.

## Conflict of Interest

The studies reported here were funded by Novartis Pharma AG, the current employer of A.K. and S.S., and the former employer of L.S.

## Supporting information

Additional supporting information may be found in the online version of this article at the publisher's web‐site.

**Figure S1.** List of IgG‐derived peptides identified by MAPPs from IVIg‐loaded DCs. Only peptides sharing at least nine amino acids with the reference human IgG1 antibody sequence are shown.
**Figure S2.** List of IgG‐derived peptides identified by MAPPs from fresh human PBMCs. Only peptides sharing at least nine amino acids with the reference human IgG1 antibody sequence are shown.
**Figure S3.** Number of identical peptides derived from Tregitopes 167 and 289 identified by MAPPs. For detection control, 1 pmol of each Tregitope was spiked into a peptide sample isolated from mature unloaded DCs. 5.4×106 DCs were loaded with Tregitope “naked” peptides at 0.39pM (donors T1 to T3) or at 3.9pM (donors T4 to T8).
**Figure S4.** List of IgG‐derived peptides identified by MAPPs from mouse splenocytes. Only peptides sharing at least nine amino acids with the reference mouse IgG2a antibody sequence are shown.
**Figure S5.** Comparison of the quantification of peptide presentation via the peak integration method versus the database search approach. Human DCs were loaded with 14nmol/mL of OVA and IVIg was added at 14nmol/mL (2 mg/mL) for donor 1–3 and at 117 nmol/mL (17 mg/ mL) for donor 4. (A) Quantification of selected OVApeptides. (B) Quantification of selected peptides derived from well‐presented endogenous proteins, isoform of annexin A2 (ANXA2), and lysosomal alpha‐mannosidase (MAN2B1).
