# Supplementary material for: Tregitopes and impaired antigen presentation: Drivers of the immunomodulatory effects of IVIg?
Source: Immun Inflamm Dis. 2017 May 31;5(4):400–15. doi: 10.1002/iid3.167 (PMC5691310; doi:10.1002/iid3.167)

Supporting information Figure 1

	aligned sequences	ocurrences
	CQQYHDTPVTFGPGTK	1
	DGSEIYYVDSVKGRFT	1
	EGRFTLSRDNSMDTL	1
	ELTQSPPSLSASVGDRV	1
	ESGGGLVPPGGSLTLSCAAS	1
	ESGGGVVQPDKSLTLSCAAS	1
	EWVSYISSSTSSTYYADSVRG	1
	GDVLGENYADWYQQKPGQ	1
	GFTFSSYSMNWVRQAMVEPGGS	1
	GGVSGSSVTGSGFSVSDLAP	1
	GKGLEYMGFIYPGDSDTKYSPSFQG	1
	GTLVTISSASTKGPSVFP	1
	IYMVSDRASGAPDRFS	1
	KGLEWVSGISRNGGSIG	1
	KGLEWVSVITASGAS	1
	KNTIYLQMNSLRAEDTA	1
	KNTLYLQMNSLRTEDA	1
	KNTLYLQMNTLRADDTA	1
	KNYVGSVRGRFTISR	1
	KPGQSPQLLIYLGSNRASGVPD	3
	KPGQSPQLLIYLGSNRASGVPDR	1
	KPGQSPQLLIYLVSNRASGVPD	1
	KRQVQLVESGGGVVQPG	1
	KSGQAPRLLIYGASSRA	4
**Donor I1**	LAWYQHKPGRAPRLLIYGASTR	1
	LESGGGLVQDGGSLRLSCAAS	2
	LLIFGASTRAAGIPDRFS	1
	LVESGGGVVQSGGSLRLSCVASGFLFGH	1
	LVQSGGGLVKPGGSLRLSCVV	1
	NSLYLQMNSLGVEDTA	1
	NTLYLQMNSLRTEDA	1
	NTNRPSGVPDRFSGSKSGPSAS	1
	PARFSGSGSGSEFALTISTL	1
	PGKAPNLLIYDVSDL	1
	PVWPCWAPAASGSPSSQGTCPPPAS	1
	QKPGKAPNVLIYGASSLQ	1
	QRLEWMGWINAANSNTEYAQKFRGRVTI	1
	RQAPGKGLEWVSYISRTSKT	1
	SDGSFHASSSLTVKSGDE	2
	SDGSFHASSSLTVKSGDEH	1
	SGGGLVQPGGFLRLSCAAS	1
	SIYLQMNSLRAEDT	2
	SKSIAYLQMNSLKTE	7
	SLSPGERAALSCRASQSVSSSHLAW	1
	SPQLLIYLGSNRASGVPD	3
	VESGGALIQPGESLTLSCAA	1
	VQPGGSLRLSCAASGLPL	1
	VSNRPPGVSNRFSGSKSGNTA	1
	VSPISGGGGSTDFADSVKGR	1

	aligned sequences	ocurrences
	AELKKPGSSVRVSCKASGEIF	1
	APNLLISKASSLEGGVPSRF	1
	ASGFTFGSHAMNWVRQAP	2
	ATGQGLEWMGWMNPNSGNTGYAQKFQG	1
	DSKDSTYSLSSTLTL	1
	DSKDSTYSLSSTLTLSKA	6
	DSTYSLSSTLTLSK	1
	DSTYSLSSTLTLSKA	3
	EQDSKDSTYSLSSTLTLSKA	1
	ESGGGLVPPGGSLTLSCAAS	1
	ESGGGVVQPDKSLTLSCAAS	3
	GGGLVQPGTSLSLSCRVGGF	1
	GGPSVFLFPPKPKDTLMISRTPEVT	3
	GPNSDGSFHASSSLTVK	1
	GPNSDGSFHASSSLTVKSG	1
	GRIVVWGQGTLVAVSS	1
	GSGSGTDFSLTISRLEPE	1
	GSGTDFTLTLSRLEPED	1
	GTTYYPDSVKGRFSISTDTSR	1
	HDWGQGTLVTVSSGGGGSGGGGSGGS	1
	ISSSGNTKIYADSVKGRFSISRDNG	1
	KDSTYSLSSTLTL	1
	KDSTYSLSSTLTLSK	6
	KDSTYSLSSTLTLSKA	7
	KGLEWLSSISSTSTYIY	1
	KNTLYLQMSSLRVED	1
**Donor I2**	KPSNTKVDKRVEPK	1
	KTYADFVKGRFTISRDN	1
	LEPEDFALYYCQQYGDSTVTFGQG	1
	LVATGGGLIQPGGSLRL	1
	MQLVQSGGEVMKPGSSL	1
	NRPSGVPDRFSGSKSGTSASLAITGLQAEDE	1
	NTLYLQINGLRVEDSAVYY	1
	PGGSLRLSCAASGFSLN	1
	QAPGQGLEWMGGSISIYGT	2
	QAPGQGLGWMGGIIPIFGTAN	1
	QKPGKAPNVLIYGASSLQ	1
	QKPGSSVKVSCKASGGAFS	2
	SASMGSPSVLSSSPSTSGASTTG	1
	SATYYCQQYNNWPPWTFG	1
	SDGSFHASSSLTVK	1
	SDGSFHASSSLTVKS	1
	SDGSFHASSSLTVKSG	2
	SDGSFHASSSLTVKSGD	1
	SDGSFHASSSLTVKSGDE	2
	SDGSFHASSSLTVKSGDEH	1
	SGTDFTLTLSRLEPE	2
	SGTDFTLTLSRLEPED	2
	SKDSTYSLSSTLTLSKA	4
	TGGGLVQPGGAMKLSCV	1
	TISRDDSTSTLYLQMK	1
	VESGGGLAQPGGSLTVSCVA	1
	VQLVQSGAEVKKPGDSVKV	1
	YGMSWVRQAPEKGLEWVSSITGSGGSTY	1

	aligned sequences	ocurrences
Donor I3	AADTAVYYCARGNAMRGGLTY	1
	AIVSSLGKAIVSSLGKAIVSSLGKAIVS	1
	DNSENTLFLQMDNLRAEDTAMYYC	1
	DTMNLQMNSLRAEDTA	1
	FGAGTKLELKRADAAPTVS	1
	FTISRDDDNNSLYLQMNSL	1
	GNMLYLQMNSLQTEDTAV	1
	GQGTLVAVSSASTKGPS	2
	GQGTLVLVSSASTKGPS	1
	GQGTLVSVSSASTKGPS	1
	GQGTLVTVSSASTKGPS	1
	KIRPSGVPDRFSGSIDTSSN	1
	KNSIYLQMNSLRAEDT	1
	KNSLYLQMDSLRAGDTA	1
	KNTIYLQMNSLRAEDTA	1
	KNTLYLQMNSLQIDDT	1
	KNTLYLQMSNLRAEDTA	1
	KPGQSPQLLIYLGSNRASGVPD	1
	KSLYLQMNSPRAEDTA	1
	MSRDNSKNTVYLQMNSL	1
	NKTLFLQMNSLRAEDT	1
	NNRPSGIPSRFSGSRSG	1
	NSIYLQMNSLRAED	1
	NSIYLQMNSLRAEDT	4
	NSIYLQMNSLRAEDTA	2
	NSIYLQMNSLRVEDT	3
	NSLFLQMNSLRAEDT	1
	NSLYLQMNSLKTEDT	1
	NSLYLQMNSLRADDT	1
	NSVYLQMNSLRAEDT	1
	NTIYLQMNSLRAED	3
	NTIYLQMNSLRAEDT	3
	NTIYLQMNSLRAEDTA	2
	NTLYLNMNSLRAEDT	1
	NTLYLQMNSLKTEDT	2
	NTLYLQMNSLRADDT	1
	NTVYLQMNSLRAED	1
	NTVYLQMNSLRAEDT	1
	NTVYLQMNSLRAEDTA	1
	PAVLQSSGLYSLSSVVTVPSSSLGTQ	4
	PGASVKISCKASGYTL	1
	QGIMVIVSSASTKGPS	1
	QGTLVLVSSASTKGPS	1
	QGTLVTVSSASTKGPS	1
	QNSLYLQMNSLRDED	1
	QNTLYLKMNSLRAEDT	1
	QNTLYLKMNSLRAEDTA	1
	RTPEVTCVVVDVSHEDPE	1
	SKNDFSLNLSSVTAADTAV	1
	SNLHNGVPSRFSGSGSR	1
	SPQLLIYLASNRASGVPD	1
	SPQLLIYLGSNRASGVPD	3
	SRDESRTTLFLQMNSLKI	1
	TPEVTCVVVDVSHEDPE	1
	TTLYLQMNSLRAEDT	1
	TTLYLQMNSLRTEDT	1
	YDVSNLEIGVPSRFSGSGSGT	1
	YEKHKVYACEVTHQG	1
	YHDAFDIWGQGTVVTVSS	1
	YVSSFGTTTHYADSVKGRFTVS	1

	aligned sequences	ocurrences
Donor I4	AASGFPFSSYAMSWVRQGQG	1
	ADTAVYYCARGYSVSHYYFDSWGQG	1
	ALEIVMTQSPATLSLSPG	1
	DSKDSTYSLSSTLTL	1
	DSKDSTYSLSSTLTLSK	3
	DSKDSTYSLSSTLTLSKA	6
	DSTYSLSSTLTLSK	1
	DSTYSLSSTLTLSKA	3
	DSVKGRFTISRDDPKNTLF	1
	EQDSKDSTYSLSSTLTLSKA	2
	FLDLWGQGTLVTVSAPS	1
	GISERLSASRSGNTASLTIAG	1
	GKGLEWVSLITW	2
	GQPPRRLIYQVSNRDSGVPAR	1
	GSGSGTDFTLSISSLQPGDFATY	1
	GSGTDFTLTLSRLEPED	1
	HLVESGGDLVKPGGSLRLSCAASGFNF	1
	KDSTYSLSSTLTLSK	2
	KDSTYSLSSTLTLSKA	8
	KNTLYLQMSSLRVED	1
	KSGNTATLTISGTQTVDE	1
	LESGGGLVQDGGSLRLSCAAS	1
	NIGSKTVNWYQQLPGTAPKLLIYTN	1
	PEDFVTYYCQQYNSYPRTFGQ	1
	PGGSLRLSCAASGFSLN	1
	RDNGRNVVSLQMSSLR	1
	RPSGIPDRFSGSGSGNRASLTI	1
	RQHPGGGLEWIGYISYSG	1
	SDGSFHASSSLTVK	2
	SDGSFHASSSLTVKS	1
	SDGSFHASSSLTVKSG	2
	SDGSFHASSSLTVKSGD	1
	SDGSFHASSSLTVKSGDE	2
	SGTDFTLTLSRLEPED	2
	SIGDRVSITCKASQVVGSAVA	2
	SKDSTYSLSSTLTLSK	1
	SKDSTYSLSSTLTLSKA	5
	SSGLYSLSSVVTVPS	1
	SSGLYSLSSVVTVPSS	2
	VQLVQSGAEVKKPGDSVKV	1
	VQPGGSLRLSCAAPGFIFSTY	1
	WIAWVRQMPGKGLEWMGSI	1
	YSSTWYAFDVWGQGTMV	1

	aligned sequences	ocurrences
Donor I5	AAAAAAAAASSGALGALGSLVKSEP	1
	AASGSSAGGSSRGGGSGSGASDLGAGSKKSP	1
	AKDRSGITASGTPDWGQGTQ	1
	APGKGLEWVGRIKTK	1
	ASSLQGGVPLRFSGSGSGTDF	1
	CQQYHDTPVTFGPGTK	1
	DNSKKTVFLQMNSLKAD	1
	DSKDSTYSLSSTLTL	1
	DSKDSTYSLSSTLTLSK	3
	DSKDSTYSLSSTLTLSKA	8
	DSTYSLSSTLTLSK	2
	DSTYSLSSTLTLSKA	2
	ELTQSPGTLSLSPGERATF	1
	EQDSKDSTYSLSSTLTLSKA	1
	ESGGGLIKPGGSLGLSCSASGFT	1
	ESGGGVVRPGGSLRLSCAPSGFP	1
	GGSLRLSCAASGFTFTTSAMSW	1
	GPNSDGSFHASSSLTVK	2
	GTKVDNKRTVAAPSVF	1
	IQVLESGGGLVRPAGSLRLSCA	1
	KDSTYSLSSTLTLSK	3
	KDSTYSLSSTLTLSKA	8
	KGLEYVSAISTNGSI	1
	KGRFTISKNNAKN	1
	LRLSCEASGFSFSGYSMG	1
	LSGSSSGNTATLTISGTQAMDE	1
	LSSVTAADTAVYYCARVGW	1
	MTQSPSSLAMSVGQKVTMS	1
	NSAVAAAAAAAAASSGALGALGSLVKSEPS	1
	PDRFSGSKSANAASLTVSGLQ	1
	PGSAPTTMIYEDNQRPSGVPD	1
	PSGVPDRFSGSKSGTSASLAISGLQSGDEA	1
	QAPGKGLDWVSVIGSTGGQ	1
	QEDSKDSTYSLSSTLTLSKA	2
	QESGPGLVKPSGTLSLICSVSGGSVS	1
	QLVQSGGGLVQPGGPLRLSC	1
	QVQLVESGGGVVQPGRSLILS	1
	RDRLGSSVTMPDSWGQGTLV	1
	RFSGSNSGNTATLTITSVAAG	1
	RMKVAGGNIWGQGTIVTVSA	1
	RQAPGKGLEWVSAIAGSNGRTY	1
	SDGSFHASSSLTVK	2
	SDGSFHASSSLTVKS	1
	SDGSFHASSSLTVKSG	1
	SDGSFHASSSLTVKSGDE	1
	SENTLYLQMNSLRG	1
	SGGGVVQPGRSLRLPCAASGFIF	1
	SGTDFTLTLSRLEPE	1
	SGTDFTLTLSRLEPED	2
	SGTDFTLTLSRLEPEDF	1
	SKDSTYSLSSTLTLSK	1
	SKDSTYSLSSTLTLSKA	5
	SPGEGLEWVSSISSSADSIFYS	1
	SQSVRSKLAWYQQKP	1
	TAMVLLFDYWGQGTLV	1
	TLSRDISKNTLFLHM	1
	VESGGGAVQPGRSLRLSCS	1
	VESGGGVVQPGRSLRLSCAVS	1
	WGGGTTVTVSSASTKGP	1

	aligned sequences	ocurrences
Donor I6	APNLLISKASSLEGGVPSRF	1
	DNWGQGILVTVASPSTKGPS	1
	DSKDSTYSLSSTLTL	1
	DSKDSTYSLSSTLTLSK	6
	DSKDSTYSLSSTLTLSKA	4
	DSTYSLSSTLTLSK	2
	DSTYSLSSTLTLSKA	1
	EQDSKDSTYSLSSTLTLSKA	2
	ETGGGLVQPGGAMKLSCVTSGFT	1
	GLEWMGRINPTDGGAFYAQKFYG	1
	GLEWVWVSAITGNGGRTY	1
	GPNSDGSFHASSSLTVKSGD	1
	GSGTDFTLTLSRLEPED	1
	GTQTYICNVNHKPS	1
	KDSTYSLSSTLTL	2
	KDSTYSLSSTLTLSK	4
	KDSTYSLSSTLTLSKA	12
	KGLEYVSAISTNGSI	1
	KNTLYLQMNSLKVEDTA	1
	KNTLYLQMSSLRVED	1
	KSGNTASLTISGLQAVDE	1
	KSGTSASLVIRGLQSED	1
	KSSLYLQMNSLRVE	1
	KSVYLQMKSLRAEDT	1
	LRAEDTGVYYCARAPAPGTSDYWGQG	1
	LRTGDTAVYYCAKDKS	1
	LTSDDTAVYYCAKDGGAAPN	1
	LTSVTAADTAVYYCAREGRGR	1
	MQLVQSGGEVMKPGSSL	1
	PSGVPDRFSGSKSGTSASLAINGLQ	1
	QEDSKDSTYSLSSTLTLSKA	1
	QGTTVTVSSASTKGPSV	1
	QLQESGPGLVKSSETL	1
	RADRTMTSDSAGLDYWGQG	1
	RFSGSGSGTDFTLTIRSLQ	1
	RGGAGAWLGGPAASLSPPKGS	1
	RPSGVADRFSGSKSGTSASLAISGLQSE	1
	SDGSFHASSSLTVK	1
	SDGSFHASSSLTVKS	1
	SDGSFHASSSLTVKSG	2
	SDGSFHASSSLTVKSGD	1
	SDGSFHASSSLTVKSGDE	2
	SGAPDRFSGSKSGTSASLVI	1
	SGTDFTLTLSRLEPED	2
	SGTDFTLTLSRLEPEDF	2
	SKDSTYSLSSTLTLSK	2
	SKDSTYSLSSTLTLSKA	7
	TGGGLVQPGGAMKLSCV	1
	TISRDNSGNTLYLQMNSLRG	1
	TTKYNPSLDGRLTISRDTSKEQFY	1
	VEDTAVYFCAREGFYSG	1
	VESGAEVKKPGSSVKVSCKASGGTFSR	1
	VESGGGLAQPGGSLTVSCVA	1
	VQLLESGGGLVTSGGSLR	1
	VQLLESGGGSVQPGGSLRLS	1
	VQLVESGGGLVKAGGSLILS	1
	WSGYLGGMDVWGQGTTVTVSSP	1
	YKPGKAPKRLIHDGSRSEKGVPR	1
	YQQLPGKAPKLLIYSDNLR	1
	YSDRPSGIPERFSGSNSG	1

	aligned sequences	ocurrences
	ADDTATYYCAGLSGYDLISGSSIG	1
	AVSGSGLSTYYADSVRGRF	1
	DGSFFLYSKLTVDK	7
	DGSFFLYSKLTVDKS	7
	DGSFFLYSKLTVDKSR	2
	DLWGRGVLITVSSASTKGP	1
	DNAKKSLFLHMNGLRAE	1
	DPIGDKAIDVWGQGTMVFVS	1
	DSDGSFFLYSKLTVDK	1
	DSDGSFFLYSKLTVDKS	5
	DSDGSFFLYSKLTVDKSR	10
	EAPHLLIYAASNLQSGVP	1
	EGSLRLSCAASGFTFSSYAMSW	2
	FSASKSGTSVSLAITGLQAE	1
	FSGSLLGDKGALTLSGAQ	1
	GAKSGTSASLAIAGLQAE	1
	GESLRLSCAASGFSFDSYWMSW	1
	GESLRLSCAASGFTFSSFAMSW	1
	GESLRLSCTASGFPFDTYAMS	1
	GGVDDIQLTQSPAIMSASPG	1
	GIPERFSGSNSGNTATLTISRVEVG	1
	GLEWVSVIRGSGGSASYADSVK	1
	GSKSGNTASLAISGLLPE	1
	GVQCRVQLVESGGGVVQPG	1
	IRVWGQGTLVTVSSAPTK	2
	IYYKDSVKGRFTISRDNSK	1
	KASNLHNGVPSRFSGSGSR	1
	KDSTYSLSSTLTLSKA	1
	KGRFTVSRDNSGNTLYL	1
**Donor I7**	KGTTVTVSSGSASAPTLF	1
	LDSGGGLVQPPGGPWDSPVQPL	1
	LVESGGGVVQPGGSLTLSCAAT	1
	MNSLRVEDTAVYYCVRDRERRGSEYH	1
	PGGSRTLSCAASGLSFSSYAMTW	1
	PGSSAKVSCKTSGGAFTPY	1
	QGTLDTVSSASTKGPSVFP	1
	QQKPGQSPVLVIYK	1
	QVQLVESGGGLVEPGGSVR	1
	RATGIPARFSGSGSGTDFTIT	1
	RGVLCQVQLVESGGGVVQPG	1
	RQAPGKGLEWVSIISGSGA	1
	SDGSFFLYSKLTVDK	4
	SDGSFFLYSKLTVDKS	10
	SDGSFFLYSKLTVDKSR	3
	SGGGVVQPGRSLRLSCAGSGLTFERY	1
	SKNTLYLHMNSLLAE	1
	TAPKLLIYSGNQRPSG	1
	TFYMELSSLTSEDTATYYCATDGTPGGEMGSW	2
	TGTAFMQMSSLTSDDTAMFYCAMEGQPTD	1
	TLKESGPTLVKPTQTFTLTC	1
	TVNWGQGTLVTVSSGSASAPP	1
	VPDRFSGTILGNKAALTI	1
	VSDISGSGGGRNYADSVKGR	1
	WGQGTLVTVSSGSASASTLFP	1
	WVRQAPGQGLEWVANIRQDGS	1
	YCARRMAVAGSDAFDIWGQGTMV	1
	YGMNVWGHGTAVTVSSAS	1
	YKASSLMSGVPSRFSGSGSGT	1
	YQGKAGKAPKMLISDAVT	1

	aligned sequences	ocurrences
	FAVYFCQQYGTSPRPFGQ	1
	FTISRDDDNNSLYLQMNSL	1
	FTISRDNSTNTLYLQMNSL	1
	FTVSRDDSKNSLYLQMNSL	1
	GPNSDGSFHASSSLTVKSGD	1
	KSLYLEMNSLRAED	1
	LRDEDTAVYYCVRGT	1
	MSWVRQAPGKGLEWVSSLSHSGDSAYYADS	1
	NKTLYLQMNSLRAEDT	1
	NSIYLQMNSLRAED	1
	NSIYLQMNSLRAEDT	3
	NSIYLQMNSLRAEDTA	1
	NSIYLQMNSLRVEDT	2
	NTIYLQMNSLRAED	2
**Donor I8**	NTIYLQMNSLRAEDT	3
	NTIYLQMNSLRVEDT	3
	NTLYLQMNSLKTEDT	1
	NTLYLQMNSLRADDT	1
	NTVYLQMNSLRAED	1
	NWYQQKPGKAPKVLVHTASTL	1
	PARFSGSLLGDKAALTLSGVQ	1
	QCEVQLLESGGGLVQPGGSR	1
	QNSLYLQMNSLRDED	1
	RAYADSVRGRFTISRDNVT	2
	SDGSFHASSSLTVKSGD	1
	SLRLSCVAYGFTFEK	1
	SPQLLIYLGSNRASGVPD	1
	TVTTYYYYGMDVWGQGTTVTVSSGS	1
	YYTSSFTFGQGTKVDIK	1

	aligned sequences	ocurrences
Donor I9	ADSVKGRFTIYRDKSNNMVY	1
	DSKDSTYSLSSTLTL	1
	DSKDSTYSLSSTLTLSK	1
	DSKDSTYSLSSTLTLSKA	1
	DSTYSLSSTLTLSK	1
	DVWGQGTMVTVSSASTKGPSVFP	1
	EVQLVQSGAEVKKPGATVKISCKASGYTFSD	1
	FSVYAMSWVRQGPGK	2
	GGSLRLSCVASGFSLDDY	1
	GPNSDGSFHASSSLTVK	1
	GPNSDGSFHASSSLTVKSG	1
	GPNSDGSFHASSSLTVKSGD	1
	KDSTYSLSSTLTLSK	4
	KDSTYSLSSTLTLSKA	4
	LNCYVSGFHPSDIEVD	1
	MYLQMNSLKTEDTAVY	1
	NQGDFSLSVRVGDQVT	1
	RNTLFLNMNSLRPEDTAV	1
	RSLDGFDVWGQGTMVTVSSGSASV	1
	SDGSFHASSSLTVK	2
	SDGSFHASSSLTVKS	1
	SDGSFHASSSLTVKSG	2
	SDGSFHASSSLTVKSGD	1
	SDGSFHASSSLTVKSGDE	2
	SGLYSLSSVVTVPSS	1
	SGTDFTLTLSRLEPE	1
	SGTDFTLTLSRLEPED	1
	SNRPSGVSNRFSGSKYGN	1
	SYTTYGISWVRQAPGQGLE	1

	aligned sequences	ocurrences
Donor I10	AASGFIFGSYGMNWIR	1
	AASGFIFNNYAMNWVR	2
	AASGFIFSGHWMHWVR	3
	AASGFIFSNHGMHWV	1
	AASGFIVSSYGMTWVR	1
	AASGFSFSDFALSWVR	1
	AMLQLTTGGETQQQIQAAMGFKIDDKGMA	1
	APRLIIYDASNRATGIPA	3
	APRLLIYGGSSRATGV	1
	APSSKSTSGGTAALGCLVKDY	1
	CAASGFSFGNYAMTW	1
	DTPGLPKIYAREGPDPYSVRVEDGAGTAGGTVP	1
	ENTVFLQMNSLKTEDT	1
	ESGPGLVTPSGTLSLTCAV	1
	FSGSGSGTDFISSLQPE	1
	GGEVAKPGASVKVSCKAAGYIF	1
	GKGLEWVSGISGTGGSTNSA	2
	GKGLEWVSYISSGS	1
	GPGTKVDAKRTVAAPSVFI	1
	GRFSGSGSGTDFTLTISGLQS	1
	GSGAASGTVVAAAAGGPGPGAGGVAAAGPAP	1
	GSGTSSPSSFTGSPGPASPGIGTSSPGSLGGSPG[illegible]	1
	ISRSSSYIYYADSVKGRLT	1
	KDTLYLQMNSLKIE	1
	KGRFTIPRENPKNT	1
	LAWYQHKPGRAPRLLIYGASTR	1
	LEWLSGISTSGSNVNLADSV	1
	LSLTSVTAADTAVYYCAR	1
	LSSVTAADTAVYYCAKGAT	1
	LSSVTAADTAVYYCAKQ	1
	LSSVTAADTAVYYCARMGK	2
	MAEVQLLESGGGLVQPGGSLT	1
	MSVVASKNQFSLKLISVTAADTA	1
	NSIYLQMNSLRAEDT	1
	NSLYLQMTTLRAEDT	1
	NTLYLQMDSLRAGDTA	1
	NTLYLQMNSLKVEDT	3
	NVENRGSRSHSPAHASNVGSPLSSPLSSM	1
	PERFSGSSSGTMATLTISGAQM	1
	PPLGSSPGSPGSQSLSSGETVPIP	2
	QAPGKGPEWVASISGS	1
	QSPSSLSASVGDSVTITCRAT	3
	QVQLQQWGAGLLKPSETLSLTCVVH	1
	RASQGISSRLAWYQQKPGKAP	1
	RDNSKNTLFLHMNSLRAE	1
	RPSGIPERVSGSKSGNTATLT	1
	RQPAGKGLEWIGRIFASG	1
	RSTISRDNSKNVLFLQ	1
	RYTDSVRGRFTVSRDTSK	2
	SAGSSGVISFGGTSLSAGKTSFSFG	2
	SCGISGIGSGFSSAFGGSSSGGNTG	1
	SDGSFFLYSKLTVDKSR	1
	SGGDSVQAGESLRLSCTGAGF	1
	SGGGLVQPGGSLRLSCAASHF	1
	SGYIFATDWIGWVRQMPG	2
	SLTAADTVGYFCARGLSGTAAR	1
	SQFSPAGSLHSPVGVCSSTGNSHS	1
	SSATVAAASATTAASSSLATP	1
	TLKESGPTLVKPSQTLTLT	1
	TSQNVSSNFAWYQQKPGQTPR	1
	TTTATVSFVVTAGGGRGPAAP	2
	TVSSVTAADTAVYYCARVGNG	1
	VQYEVQLLESGGGLVQPG	1
	VTERPSGVPDRFSGSKSGNTASLTISGLQAD	1
	WGRGTTVTVSSASTQSPSV	1

Supporting information Figure 2

	aligned sequences	ocurrences
Donor P1	DSKDSTYSLSSTLTLSK	1
	DSKDSTYSLSSTLTLSKA	1
	KDSTYSLSSTLTL	1
	KDSTYSLSSTLTLSK	3
	KDSTYSLSSTLTLSKA	2
	KGLEWVSLITGDGGY	1
	QSGGVVVQPGDSLRLS	1
	SDGSFHASSSLTVK	1
	SDGSFHASSSLTVKS	2
	SDGSFHASSSLTVKSG	2
	SDGSFHASSSLTVKSGD	1
	SDGSFHASSSLTVKSGDE	1
	SKDSTYSLSSTLTLSKA	1
	WVSYISSSGGTIYYANSV	1
Donor P2	GDWGQGTLVTVSSAS	1
	KSLYLEMNSLRAEDTA	1
	NSIYLQMNSLRAED	1
	NSIYLQMNSLRAEDT	1
	NTIYLEMNSLRAEDT	1
	NTIYLQMNSLRAED	1
	NTIYLQMNSLRAEDT	2
	NTIYLQMNSLRVEDT	2
	NTLYLEMNSLRVEDT	1
	NTLYLQMSNLRAEDTA	1
	SIYLQMNSLRAED	1
	SLYLQMTTLRAEDT	1
	TVYLQMNSLRAED	1
Donor P3	KSLYLEMNSLRAEDT	1
	KSLYLEMNSLRAEDTA	1
	NTIYLQMNSLRAEDT	2
	NTIYLQMNSLRAEDTA	1
	NTVYLQMNSLRAEDTA	1
	VSDISGSGGSTNYADSVE	1
Donor P4	APKLLIYKASSLE	1
	KPGKAPKLLIYKASSLES	1
	LPGTAPKLLIHNNNQRPSG	1
	LPGTAPKLLIYSNNQGPSGV	1
	LPGTAPKLLIYSNNQRPS	2
	LPGTAPKLLIYSNNQRPSG	2
	TAPKLLIYSNNQRPSG	2
Donor P5	ATLSCRASQSVSINLAWYQ	1
	DGSFFLYSKLTVDKS	1
	DGSFFLYSKLTVDKSR	1
	KDSTYSLSSTLTLSK	1
	KDSTYSLSSTLTLSKA	1
	NSIYLQMNSLRAED	1
	NSIYLQMNSLRAEDT	1
	NTIFLQMNSLRAEDT	2
	NTIYLEMNSLRAEDT	1
	NTIYLQMNSLRAEDT	2
	NTIYLQMNSLRAEDTA	2
	SGGGVVQPGKSLTLSCAVSG	1

	aligned sequences	ocurrences
Donor P6	APKLLIYKASSLE	1
	DSKDSTYSLSSTLTLSKA	1
	DSTYSLSSTLTLSKA	1
	KDSTYSLSSTLTLSK	5
	KDSTYSLSSTLTLSKA	3
	LPGTAPKLLIYSNNQRPS	1
	SDGSFHASSSLTVK	1
	SDGSFHASSSLTVKS	2
	SDGSFHASSSLTVKSG	3
	SDGSFHASSSLTVKSGD	1
	SRFSGSGSGAEFTLTISG	1
	TAPKLLIYSNNQRPSG	1
Donor P7	AERYLTISSLQSEDE	2
	AKNSIYLQMNSLRAEDT	1
	APRLIIYDASNRATGIPA	1
	ARFSGSKSGSSATLAISGLQ	1
	DSKDSTYSLSSTLTLSK	2
	GKGLEWVSVIYSGGSSYYADS	1
	KDSTYSLSSTLTLSK	2
	KNTLYVQMNSLRAEDT	1
	KPGQAPVLVIYGKNNRPSG	1
	KSLYLDMTSLRAEDT	1
	KVQWKVDNALQSGNS	1
	LVKDYFPEPVTVSWN	1
	LVKDYFPEPVTVSWNSG	1
	NMVYLQMNSLRAEDT	1
	NSIYLQMNSLRAEDT	1
	NSLYLQMDSLRAEDT	1
	NTIFLQMNSLRAEDT	2
	NTIYLQMDSLRAEDT	2
	NTIYLQMDSLRAEDTA	1
	NTIYLQMNSLRAED	1
	NTIYLQMNSLRAEDT	4
	NTIYLQMNSLRAEDTA	1
	NTLYLQMESLRAEDT	1
	RDNSKDTLYLQMGSLR	1
	SGREFILTISSLQSEDL	1
	TLYIQMNSLRAEDT	1
	VKDYFPEPVTVSWNSG	1

Supporting information Figure 3

A **Peptide processing of Tregitope 167**

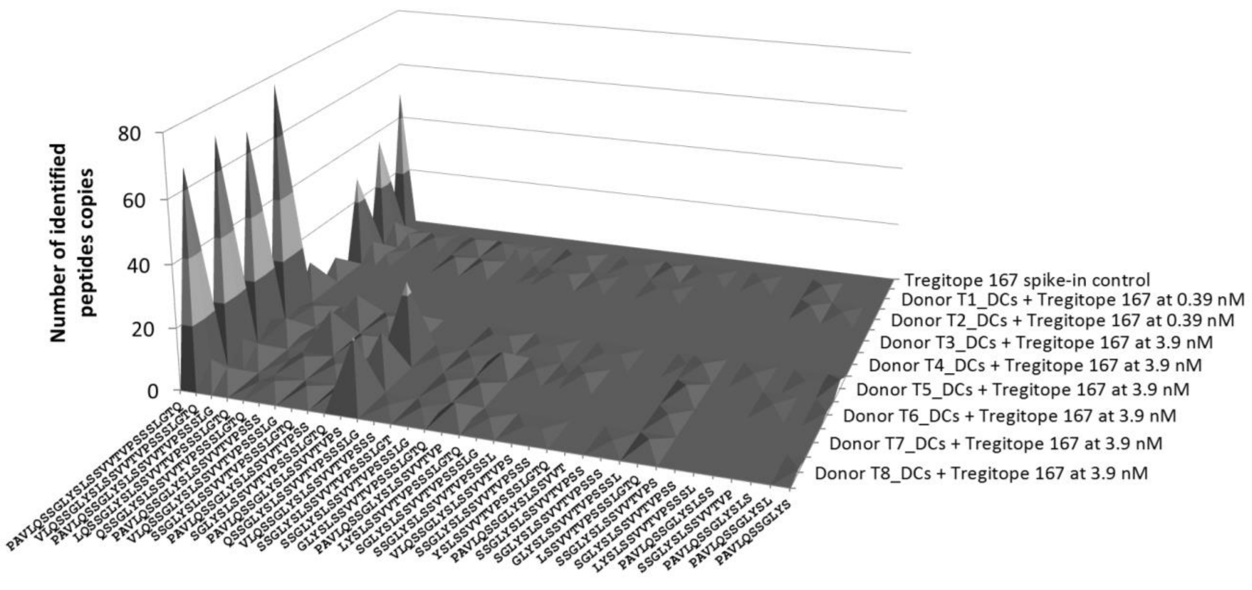

B **Peptide processing of Tregitope 289**

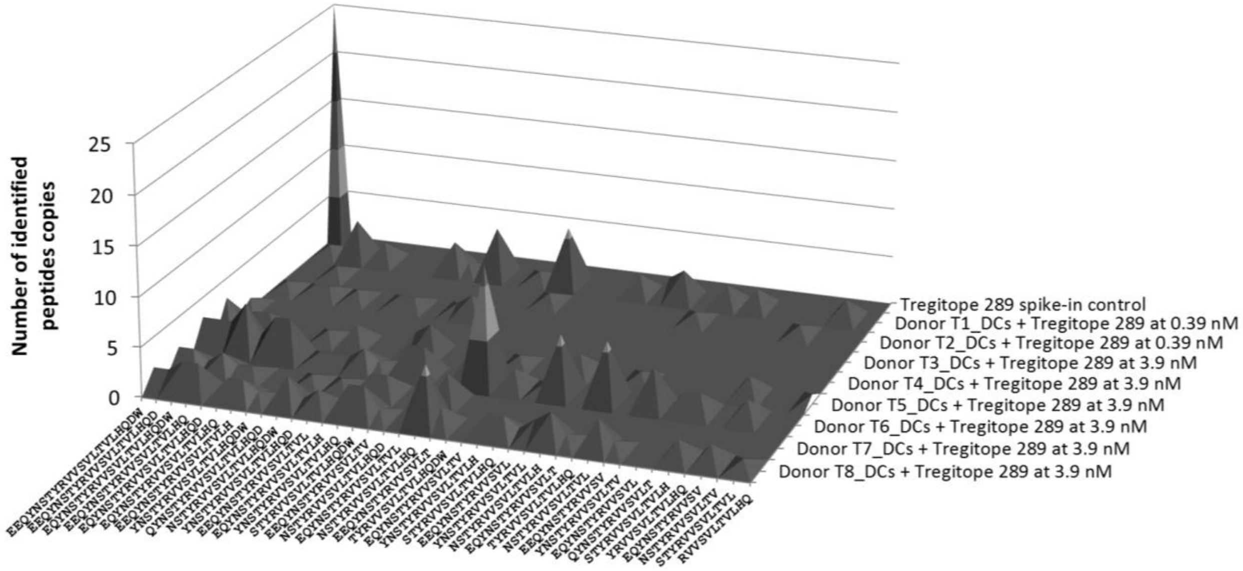

Supporting information Figure 4

	aligned sequences	ocurrences
mouse 1	DIQMTQSPASLSASVG	1
	DIQMTQSPSSLSASLG	4
	DKSSSTAYMQISSLTSEDS	1
	DQDSKDSTYSMSSTLTLTK	3
	DSKDSTYSMSSTLT	1
	DSKDSTYSMSSTLTLT	1
	DSKDSTYSMSSTLTLTK	2
	ELYHYSSSVITSSST	2
	GQPAENYKNTQPIMDTD	1
	GQPAENYKNTQPIMDTDG	4
	GSGRDYSFSLSNLEPED	1
	GSGTSYSLIISSMEAEDA	1
	GSYFVYSKLNVQKSN	1
	GVPDRFSGSGSGTDFTFTIS	1
	KDSTYSMSSTLTLTK	1
	KPDGTVKLLIYYTSSLHSGVPS	1
	KPSSTAYMQISSLTSEDS	1
	KWKALYTDPKSSETAA	1
	LQQPGADLVKPGASVTLSCKAS	1
	NGQPAENYKNTQPIMDTDG	1
	QPAENYKNTQPIMDTD	3
	QPAENYKNTQPIMDTDG	4
	QPAENYKNTQPIMDTDGS	1
	QSPSYLAASPGETIT	2
	SCKASGYTFTNYWLGWV	1
	SKDSTYSMSSTLTLTK	2
	SPAPAALLGPSSVQSPP	1
	SPKLWIYSTSNLASGVPA	2
	SPKLWIYYTSNLAPGVP	1
	SPKLWIYYTSNLAPGVPA	2
	SPKPWIYATSNLASGVPA	1
	SPKTLIYYATSLADGVPS	2
	SPKTWIYATSNLASGVPA	2
	SSATYMQLSSLTSEDS	1
	SSGVHTFPAVLQSDLY	2
	SSGVHTFPAVLQSDLYT	1
	SSTAYMELSSLTSEDS	1
	SSTAYMQISSLTSEDS	1
	SYFVYSKLNVQKSN	2
	TDQDSKDSTYSMSSTLT	1
	TELYHYSSSVITSSST	1
	TPSSPSYSPSSPSYSPTSPK	1
	TQTPAVMSASLGERVTMTCT	1
	VDKSSSTAYMQISSLTSEDS	1
	VKLLIYYTSRIHSGVPS	2
	VPARFSGSGSGTSYS	1
	WMSWVPQAPGKGLEW	1
	WPSQTVTCNVAHPAS	4
	WPSQTVTCNVAHPASS	3
	WPSQTVTCNVAHPASST	7
	WPSQTVTCSVAHPAS	1
	WPSQTVTCSVAHPASST	11
	YKNTEPVLDSDGSYFMYSKL	1
	YKNTEPVLDSDGSYFMYSKLRVE	1

	aligned sequences	ocurrences
mouse 2	ATVTADKSSSTAYMQLTSLTSED	1
	DGQAPENYKNTQPIMDTDG	1
	DGSYFVYSKLNVQKSN	2
	DIQMTQSPASLSASVG	1
	DIQMTQSPSSLSASLG	3
	DLYTLSSSVTVTSSTWPS	1
	DQDSKDSTYSMSSTLTLT	1
	DQDSKDSTYSMSSTLTLTK	2
	DSKDSTYSMSSTLTL	1
	DSKDSTYSMSSTLTLT	1
	DSKDSTYSMSSTLTLTK	2
	ELYHYSSSVITSSST	2
	FEGKATLTVDTSSSTAY	1
	GQPAENYKNTQPIMD	1
	GQPAENYKNTQPIMDTD	2
	GQPAENYKNTQPIMDTDG	4
	GSGRDYSFSLSNLEPED	2
	GSYFVYSKLNVQKSN	1
	GSYFVYSKLNVQKSNWE	1
	KDSTYSMSSTLTLTK	1
	KPDGTVKLLIYYTSSLHSGVPS	1
	KPGNIPKLLIYKASNLHTGVPS	1
	KWKALYTDPKSSETAA	1
	LSSGVHTFPAVLQSDLY	1
	LSSGVHTFPAVLQSDLYT	1
	NGQPAENYKNTQPIMDTDG	1
	QPAENYKNTQPIMDTD	3
	QPAENYKNTQPIMDTDG	6
	QPAENYKNTQPIMDTDGS	2
	QPPKLLIYAASNVESGVPA	1
	SDLYTLSSSVTVTSSTWPS	1
	SGPELVSPGASVKMSCKASGY	1
	SGSGRDYSFSISDLEPEDIA	1
	SKDSTYSMSSTLTLTK	1
	SPAPAALLGPSSVQSPP	1
	SPKLWIYSTSNLASGVPA	2
	SPKLWIYYTSNLAPGVPA	2
	SPKRWIYDTSKLASGVPA	1
	SPKTLIYYATSLADGVPS	2
	SPKTWIYATSNLASGVPA	1
	SSGVHTFPAVLQSDLY	2
	SSTAYMQISSLTSEDS	1
	SYFVYSKLNVQKSN	2
	SYFVYSKLNVQKSNWE	1
	SYFVYSKLNVQKSNWEA	1
	TDQDSKDSTYSMSSTLT	1
	TELYHYSSSVITSSST	1
	TPSSPSYSPSSPSYSPTSPK	1
	VKLLIYYTSRIHSGVPS	1
	VPARFSGSGSGTSYS	1
	WPSETVTCNVAHPASST	1
	WPSETVTCSVAHPAS	1
	WPSETVTCSVAHPASSTT	1
	WPSQTVTCNVAHPAS	6
	WPSQTVTCNVAHPASS	4
	WPSQTVTCNVAHPASST	10
	WPSQTVTCSVAHPA	3
	WPSQTVTCSVAHPAS	6
	WPSQTVTCSVAHPASS	2
	WPSQTVTCSVAHPASST	15
	YKNTEPVLDSDGSYFMYSKL	1
	YKNTEPVLDSDGSYFMYSKLRVE	1

	aligned sequences	ocurrences
mouse 3	DIQMTQSPASLSASVG	1
	DIQMTQSPSSLSASLG	3
	DQDSKDSTYSMSSTLTL	1
	DQDSKDSTYSMSSTLTLT	1
	DQDSKDSTYSMSSTLTLTK	4
	DSKDSTYSMSSTLT	1
	DSKDSTYSMSSTLTLT	1
	DSKDSTYSMSSTLTLTK	3
	ELYHYSSSVITSSST	2
	FKDKATLTVDKSSRT	1
	GAGLVKPGASVEISCKATGYT	1
	GQAPENYKNTQPIMDTDGS	1
	GQPAENYKNTQPIMD	2
	GQPAENYKNTQPIMDTD	2
	GQPAENYKNTQPIMDTDG	5
	GSGRDYSFSLSNLEPED	2
	GSGSGRDYSFSLSNLEPED	1
	GSYFVYSKLNVQKSN	2
	GSYFVYSKLNVQKSNWE	1
	GVPDRFSGSGSGTDFTFTIS	1
	IKLLIYSTSNLNSGVPS	1
	KPDGTVKLLIYYTSSLHSGVPS	1
	KWKALYTDPKSSETAA	1
	LSSGVHTFPAVLQSDLY	1
	NGQPAENYKNTQPIMD	1
	NGQPAENYKNTQPIMDTDG	1
	QPAENYKNTQPIMDTD	4
	QPAENYKNTQPIMDTDG	4
	QPAENYKNTQPIMDTDGS	1
	QSPSYLAASPGETIT	1
	SDLYTLSSSVTVTSSTWPS	1
	SGVHTFPAVLQSDLY	1
	SKDSTYSMSSTLTLTK	1
	SLSASLGDTITITCHASQNIN	1
	SPAPAALLGPSSVQSPP	1
	SPKLWIYSTSNLASGVPA	1
	SPKLWIYYTSNLAPG	1
	SPKLWIYYTSNLAPGVP	1
	SPKLWIYYTSNLAPGVPA	2
	SPKPWIYATSNLASGVPA	1
	SPKTLIYYATSLADGVPS	1
	SSATYMQLSSLTSEDS	1
	SSGVHTFPAVLQSDLY	2
	SSTAYMQFSSLTSEDS	1
	SSTAYMQISSLTSEDS	1
	SYFVYSKLNVQKSN	2
	SYFVYSKLNVQKSNWE	2
	SYFVYSKLNVQKSNWEA	1
	TDQDSKDSTYSMSSTLT	1
	TELYHYSSSVITSSST	1
	TPSSPSYSPSSPSYSPTSPK	1
	TSNPPTFGGGTKLEIKRT	1
	VKLLIYYTSRIHSGVPS	3
	VPSSTWPSETVTCSVAHPASST	1
	VPSSTWPSQTVTCNVAHPA	1
	WMSWVPQAPGKGLEW	1
	WPSETVTCNVAHPASST	1
	WPSQTVTCNVAHPA	2
	WPSQTVTCNVAHPAS	5
	WPSQTVTCNVAHPASS	4
	WPSQTVTCNVAHPASST	12
	WPSQTVTCNVAHPASSTK	1
	WPSQTVTCSVAHPA	4
	WPSQTVTCSVAHPAS	8
	WPSQTVTCSVAHPASS	3
	WPSQTVTCSVAHPASST	19
	WPSQTVTCSVAHPASSTT	4
	YKNTEPVLDSDGSYFMYSKL	1
	YKNTEPVLDSDGSYFMYSKLRVE	1

	aligned sequences	ocurrences
mouse 4	AAALAAAAAGGTAYMGLGGLSSE	1
	DEDSKDSTYSMSSTLTLTK	1
	DGAPAPSGPPPPGTGRSSGKHS	1
	DIQMTQSPASLSASVG	1
	DIQMTQSPSSLSASLG	3
	DQDSKDSTYSMSSTLTL	1
	DQDSKDSTYSMSSTLTLT	1
	DQDSKDSTYSMSSTLTLTK	3
	DSKDSTYSMSSTLT	1
	DSKDSTYSMSSTLTLT	1
	DSKDSTYSMSSTLTLTK	1
	ELYHYSSSVITSSST	1
	EPYTELYHYSSSVITSSSTR	1
	GQAPENYKNTQPIMDTDG	2
	GQPAENYKNTQPIMDTD	3
	GQPAENYKNTQPIMDTDG	2
	GSGRDYSFSLSNLEPED	1
	GSYFVYSKLNVQKSN	3
	GVPDRFSGSGSGTDFTFTIS	1
	IPKLLIYKASNLHTGVPS	1
	KATLTVDTSSTTAYMQ	1
	KDSTYSMSSTLTLTK	1
	KPDGTVKLLIYYTSRIHSGVPS	1
	KPDGTVKLLIYYTSSLHSGVPS	1
	KWKALYTDPKSSETAA	1
	NGQPAENYKNTQPIMDTDG	1
	QPAENYKNTQPIMDTD	4
	QPAENYKNTQPIMDTDG	4
	QPAENYKNTQPIMDTDGS	1
	QPPKLLIYAASNVESGVPA	1
	QSPSYLAASPGETIT	2
	SDLYTLSSSVTVTSSTWPS	1
	SGSGSGNSYSLTISSMEAEDA	1
	SKDSTYSMSSTLTLTK	1
	SPKLWIYSTSNLASGVPA	2
	SPKLWIYYTSNLAPGVP	1
	SPKLWIYYTSNLAPGVPA	2
	SPKPWIYATSNLASGVPA	1
	SPKTLIYYATSLADGVPS	1
	SPKTWIYATSNLASGVPA	1
	SSATYMQLSSLTSEDS	1
	SSGVHTFPAVLQSDLY	2
	SSGVHTFPAVLQSDLYT	1
	SSTAYMQISSLTSEDS	2
	SYFVYSKLNVQKSN	2
	SYFVYSKLNVQKSNWEA	1
	TDQDSKDSTYSMSSTLT	1
	TELYHYSSSVITSSST	2
	TPSSPSYSPSSPSYSPTSPK	1
	VEWTNNGKTELNYKNTEPVLDSDGSY	1
	VKLLIYYTSRIHSGVPS	1
	WIGEILPGSGSTNCN	1
	WPSETVTCNVAHPASST	1
	WPSQTVTCNVAHPAS	4
	WPSQTVTCNVAHPASS	4
	WPSQTVTCNVAHPASST	11
	WPSQTVTCNVAHPASSTK	1
	WPSQTVTCSVAHPA	1
	WPSQTVTCSVAHPAS	6
	WPSQTVTCSVAHPASS	2
	WPSQTVTCSVAHPASST	15
	WPSQTVTCSVAHPASSTT	2
	YKNTEPVLDSDGSY	1
	YKNTEPVLDSDGSYFMYSKL	2
	YKNTEPVLDSDGSYFMYSKLRVE	1

	aligned sequences	ocurrences
mouse 5	ADRSSSTAYMQLSSLTSEDS	1
	DEDSKDSTYSMSSTLTL	1
	DEDSKDSTYSMSSTLTLTK	1
	DGAPAPSGPPPPGTGRSSGKHS	1
	DGSYFVYSKLNVQKSN	2
	DGSYFVYSKLNVQKSNWE	1
	DIQMTQSPASLSASVG	3
	DIQMTQSPSSLSASLG	4
	DKSSSTAYMELSSLTSEDS	1
	DKSSSTAYMQISSLTSEDS	1
	DQDSKDSTYSMSSTLT	2
	DQDSKDSTYSMSSTLTL	1
	DQDSKDSTYSMSSTLTLT	1
	DQDSKDSTYSMSSTLTLTK	4
	DSKDSTYSMSSTLT	1
	DSKDSTYSMSSTLTL	1
	DSKDSTYSMSSTLTLT	2
	DSKDSTYSMSSTLTLTK	2
	FNSKATLTVDKPSSTTYM	1
	GQPAENYKNTQPIMD	2
	GQPAENYKNTQPIMDTD	2
	GQPAENYKNTQPIMDTDG	4
	GQPAENYKNTQPIMDTDGS	1
	GSGRDYSFSLSNLEPED	1
	GSYFVYSKLNVQKSN	3
	GSYFVYSKLNVQKSNWE	1
	HVHILLANLYVVLPPSLNPIIYGVKTKQI	1
	IPKLLIYKASNLHTGVPS	1
	KDSTYSMSSTLTLT	1
	KDSTYSMSSTLTLTK	1
	KPSSTAYMQISSLTSEDS	1
	KWKALYTDPKSSETAA	1
	LSSGVHTFPAVLQSDLY	1
	LSSGVHTFPAVLQSDLYT	1
	NGQPAENYKNTQPIMD	1
	NGQPAENYKNTQPIMDTDG	2
	QPAENYKNTQPIMDTD	2
	QPAENYKNTQPIMDTDG	5
	QPAENYKNTQPIMDTDGS	2
	QSPSYLAASPGETIT	1
	SCKASGYTFTSYVMHWV	1
	SDLYTLSSSVTVPSSTWPS	1
	SDLYTLSSSVTVTSSTWPS	1
	SGVHTFPAVLQSDLY	1
	SPKLWIYSTSNLASGVPA	2
	SPKLWIYYTSNLAPGVP	1
	SPKLWIYYTSNLAPGVPA	2
	SPKTLIYYATSLADGVPS	2
	SPKTWIYATSNLASGVPA	1
	SSGVHTFPAVLQSDLY	2
	SSGVHTFPAVLQSDLYT	1
	SSTAYMQISSLTSEDS	3
	SVKMSCKASGYTFTDYT	1
	SYFVYSKLNVQKS	1
	SYFVYSKLNVQKSN	2
	SYFVYSKLNVQKSNWE	2
	SYFVYSKLNVQKSNWEA	2
	TDQDSKDSTYSMSSTLT	1
	TDQDSKDSTYSMSSTLTLTK	1
	TELYHYSSSVITSSST	1
	TPSSPSYSPSSPSYSPTSPK	1
	TSNPPTFGGGTKLEIKRT	1
	VKLLIYYTSRIHSGVPS	4
	VPARFSGSGSGTSYS	1
	VPSSTWPSETVTCSVAHPASST	1
	VPSSTWPSQTVTCNVAHP	1
	VPSSTWPSQTVTCNVAHPA	2
	VPSSTWPSQTVTCNVAHPASST	2
	VPSSTWPSQTVTCSVAHPASST	1
	WIGYINPSSGYTKYN	1
	WNGQPAENYKNTQPIMDTDG	1
	WPSQTITCNVAHPASST	1
	WPSQTITCNVAHPASSTK	1
	WPSQTVTCNVAHPA	2
	WPSQTVTCNVAHPAS	8
	WPSQTVTCNVAHPASS	6
	WPSQTVTCNVAHPASST	15
	WPSQTVTCNVAHPASSTK	2
	WPSQTVTCSVAHPA	3
	WPSQTVTCSVAHPAS	4
	WPSQTVTCSVAHPASS	5
	WPSQTVTCSVAHPASST	20
	WPSQTVTCSVAHPASSTT	2

Supporting information Figure 5

A

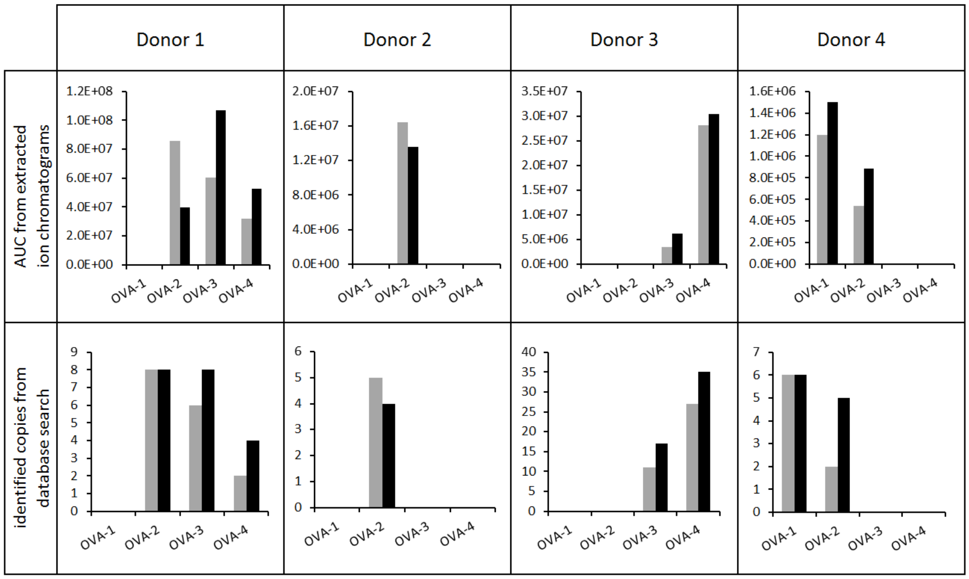

B

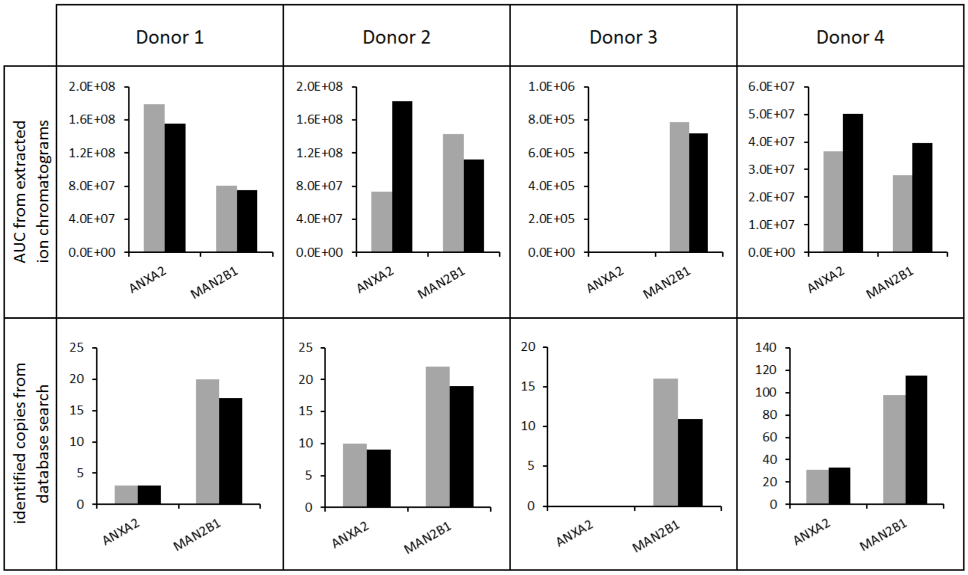

Supplement: Supplementary file 1 — Figure S1. List of IgG‐derived peptides identified by MAPPs from IVIg‐loaded DCs. Only peptides sharing at least nine amino acids with the reference human IgG1 antibody sequence are shown. Figure S2. List of IgG‐derived peptides identified by MAPPs from fresh human PBMCs. Only peptides sharing at least nine amino acids with the reference human IgG1 antibody sequence are shown. Figure S3. Number of identical peptides derived from Tregitopes 167 and 289 identified by MAPPs. For detection control, 1 pmol of each Tregitope was spiked into a peptide sample isolated from mature unloaded DCs. 5.4×10^6^ DCs were loaded with Tregitope “naked” peptides at 0.39pM (donors T1 to T3) or at 3.9pM (donors T4 to T8). Figure S4. List of IgG‐derived peptides identified by MAPPs from mouse splenocytes. Only peptides sharing at least nine amino acids with the reference mouse IgG2a antibody sequence are shown. Figure S5. Comparison of the quantification of peptide presentation via the peak integration method versus the database search approach. Human DCs were loaded with 14nmol/mL of OVA and IVIg was added at 14nmol/mL (2 mg/mL) for donor 1–3 and at 117 nmol/mL (17 mg/ mL) for donor 4. (A) Quantification of selected OVApeptides. (B) Quantification of selected peptides derived from well‐presented endogenous proteins, isoform of annexin A2 (ANXA2), and lysosomal alpha‐mannosidase (MAN2B1). [file IID3-5-400-s001.pdf]
